# Associations of Breast Cancer Treatments with One-Year Changes in Health-Related Fitness †

**DOI:** 10.3390/cancers17244026

**Published:** 2025-12-17

**Authors:** Fernanda Z. Arthuso, Ki-Yong An, Qinggang Wang, Renée L. Kokts-Porietis, Andria R. Morielli, Margaret L. McNeely, Jeff K. Vallance, S. Nicole Culos-Reed, Gordon J. Bell, Leanne Dickau, Myriam Filion, Stephanie M. Ntoukas, Jessica McNeil, Lin Yang, Charles E. Matthews, Christine M. Friedenreich, Kerry S. Courneya

**Affiliations:** 1 Faculty of Kinesiology, Sport, and Recreation, College of Health Sciences, University of Alberta, Edmonton, AB T6G 2H9, Canada; 2 Department of Cancer Epidemiology and Prevention Research, Cancer Care Alberta, Calgary, AB T2N 5G2, Canada; 3 Cancer Prevention and Screening Innovation, Primary Care Alberta, Calgary, AB T2W 3N2, Canada; 4 Faculty of Rehabilitation Medicine, University of Alberta, Edmonton, AB T6G 2G4, Canada; 5 Supportive Care and Patient Experience, Cancer Care Alberta, Edmonton, AB T6G 1Z2, Canada; 6 Faculty of Health Disciplines, Athabasca University, Athabasca, AB T9S 3A3, Canada; 7 Departments of Oncology and Community Health Sciences, Cumming School of Medicine, University of Calgary, Calgary, AB T2N 1N4, Canada; 8 Faculty of Kinesiology, University of Calgary, Calgary, AB T2N 1N4, Canada; 9 Department of Kinesiology, School of Health and Human Sciences, University of North Carolina at Greensboro, Greensboro, NC 27412, USA; 10 Division of Cancer Epidemiology and Genetics, US National Cancer Institute, Rockville, MD 20892, USA

**Keywords:** body composition, breast cancer, cardiorespiratory fitness, chemotherapy, muscle strength

## Abstract

**Background/Objectives:** Early-stage breast cancer treatments adversely affect components of health-related fitness (HRF) important for treatment tolerability, recovery, and long-term outcomes. Few studies have examined cancer treatment modality-specific effects on HRF. We examined associations of breast cancer treatment modalities, regimens, and combinations with one-year changes in HRF. **Methods:** Newly diagnosed early-stage breast cancer patients were recruited between 2012 and 2019 for the Alberta Moving Beyond Breast Cancer (AMBER) cohort study. HRF assessments were completed within 90 days of diagnosis and at one year, including cardiorespiratory fitness, muscle strength and endurance, and body composition. Analysis of covariance was used to test whether HRF changes differed between treatment modalities, regimens, and combinations. All tests were 2-sided. **Results:** A total of 1350 participants (mean [SD] age, 55.6 [10.7] years) were included. Women who received chemotherapy (*n* = 797; 59%) experienced statistically significant smaller increases in upper body strength (−1.7 kg, 95% confidence interval [CI]: −3.0 to −0.5), greater declines in lower body endurance (−118.0 kg, 95%CI: −216.6 to −19.3), and greater declines in total lean mass (−0.7 kg, 95%CI: −1.1 to −0.3), bone mineral density (−0.01 g/cm^2^, 95%CI: −0.02 to 0.00), and bone mineral content (0.04 kg, 95%CI: −0.06 to −0.02). Other treatment modalities were modestly and inconsistently associated with HRF changes. Treatment combinations that included chemotherapy had the most negative impact on cardiorespiratory fitness and body composition. **Conclusions:** Chemotherapy—either alone or in combination with other treatments—had the largest and broadest negative impact on HRF recovery in early-stage breast cancer at one-year follow-up.

## Simple Summary

While treatments for small and localized breast cancer are often curative, they can have negative effects on physical fitness. This issue matters because greater physical fitness helps patients tolerate treatment better, recover more effectively, and maintain long-term health. In this study of 1350 women with early stage and localized breast cancer, we explored the connections between different treatment modalities and combinations with changes in physical fitness over one year. Women who received chemotherapy experienced greater reductions in lower body strength, muscle mass, and bone health compared to those who did not receive chemotherapy. Other types of treatment had smaller or less consistent associations with physical fitness. Treatment combinations that included chemotherapy had the most negative impact on cardiorespiratory fitness and body composition. Understanding how different breast cancer treatments relate to physical fitness changes may help guide the development of tailored exercise programs.

## 1. Introduction

Curative treatment modalities for breast cancer include combinations of surgery, chemotherapy, radiotherapy, targeted therapy, and hormone therapy [1], that may adversely affect multiple components of health-related fitness (HRF; e.g., cardiorespiratory fitness, muscular strength, muscular endurance, and body composition) [2,3]. HRF components are important because they are strong predictors of morbidity and mortality among adults [4], including cancer patients [5]. We previously reported that lower levels of HRF in newly diagnosed breast cancer patients were associated with worse quality of life, depression, fatigue, and sleep quality [6,7]. Poor HRF is also associated with increased treatment toxicity and dose reductions, higher breast cancer recurrence risk, and worse survival [8,9,10]. Cancer patients with high physical fitness levels have a significant reduction in the risk of all-cause mortality compared to those with low physical fitness levels [5]. Structured exercise interventions can be designed to enhance or preserve components of HRF, thereby establishing HRF as a modifiable and clinically meaningful target throughout the breast cancer care continuum. Maintaining or improving HRF during the breast cancer journey is crucial for recovery, treatment tolerability, and long-term outcomes.

Evidence on how different breast cancer treatment modalities, regimens, and combinations affect multiple components of HRF remains limited. Previous studies have primarily focused on a single treatment modality and a single HRF component, such as the effects of chemotherapy or hormone therapy on body composition [11,12]. Few primary studies have investigated the effects of distinct treatment modalities [13,14,15] or combinations [16,17] on a comprehensive set of HRF. Understanding how different breast cancer treatment modalities affect distinct HRF components is critical for developing targeted, evidence-based exercise interventions during and after breast cancer treatments. Such interventions have the potential not only to improve HRF and patient well-being, but also to enhance survival [18].

The Alberta Moving Beyond Breast Cancer (AMBER) Cohort Study includes a large sample of newly diagnosed breast cancer patients and a comprehensive set of high-quality objective HRF measures. Our study contributes meaningfully by examining how different treatment modalities, regimens, and combinations are associated with distinct changes in HRF, providing a more nuanced understanding of the impact of treatment on survivorship. Therefore, the primary purpose of this paper was to determine if different breast cancer treatment modalities and regimens were associated with changes in HRF from baseline to one-year in the AMBER Cohort. We also explored the associations of different breast cancer treatment combinations with changes in HRF. Finally, we explored the impact of age and baseline fitness on the associations of chemotherapy treatment with HRF. Drawing on findings from previous prospective [13,15] and cross-sectional [14,16] studies, and given that chemotherapy is characterized by systemic and cumulative toxicities that directly impair multiple aspects of physiological capacity [19], we hypothesized that patients receiving chemotherapy and combinations of multimodal treatments that include chemotherapy would experience the greatest negative impact on HRF components, and that older and fitter patients would be affected the most.

## 2. Materials and Methods

### 2.1. Setting and Participants

The AMBER study is a prospective cohort of 1528 women residing in Alberta, Canada, newly diagnosed with histologically confirmed stage I-IIIc breast cancer, aged 18–80, and able to complete questionnaires in English between July 2012 and July 2019. Details of the study design, data collection, and a baseline description of the full AMBER cohort are available in previous publications [20,21]. Ethics review was done by the Health Research Ethics Board of Alberta: Cancer Committee (HREBA.CC-17-0576) and participants provided signed consent.

### 2.2. Data Collection

Clinical information on patients’ cancer diagnosis and treatment was abstracted from medical charts by trained study staff, including date of diagnosis, tumor stage and grade, hormone receptor status, surgery type, treatments received, and corresponding dates. Baseline sociodemographic and lifestyle behavior characteristics were obtained from a self-administered questionnaire. The Canadian Diet History Questionnaire II [22] was used to assess past year dietary intake and estimate kilocalorie intake.

The present study includes HRF measures at baseline (within 90 days of diagnosis), and one year follow-up to reflect the short-term impact of primary breast cancer treatments on HRF. Components of HRF were objectively measured following standard protocols [20]. Cardiorespiratory fitness was assessed through direct measurement of peak oxygen consumption (VO_2peak_) by the modified Bruce protocol on a treadmill [23], using an automated metabolic measurement system (TrueOne 2400, Parvo Medics Inc, Sandy, UT, USA). Muscular fitness included assessments of upper and lower body maximal strength and muscular endurance using chest press and leg press machines. Maximal strength was assessed by a predicted 1 repetition maximum (RM) using an 8–10 repetition maximum test [20]. Muscular endurance was assessed based on 50% of the predicted 1-RM for the chest press and 70% of the predicted 1-RM for the leg press and calculated as the number of repetitions x weight lifted. Body composition was measured by dual energy x-ray absorptiometry (DXA, GE Lunar Expert/Prodigy, GE Healthcare, Madison, WI, USA—Edmonton; Hologic Horizon A, Hologic Inc., Marlborough, MA, USA—Calgary) and expressed as total lean and total fat mass, lean and fat mass percentage, lean-to-fat ratio, bone mineral density, and bone mineral content. Body weight and height were assessed and used to calculate body mass index (BMI).

### 2.3. Statistical Analysis

We examined the clinical and sociodemographic data using descriptive analyses for the entire cohort and by chemotherapy treatment (yes vs. no chemotherapy). We used chi-square analyses for categorical variables and one-way analysis of variance for continuous variables for between group differences. Within-group changes in HRF were analyzed using paired *t*-tests. Analysis of covariance (ANCOVA) was used to test whether changes in HRF over one year differed between treatment modalities for surgery (mastectomy vs. lumpectomy), chemotherapy (yes vs. no), radiotherapy (yes vs. no), hormone therapy (yes vs. no), and targeted therapy (yes vs. no). For surgery, we restricted the analysis to neoadjuvant patients to ensure that we only included patients whose baseline assessment occurred before surgery. Within the chemotherapy group, we conducted exploratory analysis using ANCOVA, stratifying participants by baseline fitness levels (median split: top vs. bottom half, with the top half representing more favorable baseline fitness), age (≥60 vs. <60 years), chemotherapy regimen (anthracycline-based vs. taxane-based regimen), and relative dose intensity (RDI, <85% vs. ≥85%). To further explore associations between breast cancer treatment and HRF changes, we conducted exploratory analysis using ANCOVA on the three most common treatment combinations identified within our dataset (SCRH: surgery, chemotherapy, radiotherapy, and hormone therapy; SRH: surgery, radiotherapy, and hormone therapy; SH: surgery and hormone therapy) and on the number of therapies (≤2 vs. 3 vs. 4 vs. 5). All ANCOVA models were adjusted for relevant covariates, selected based on prior knowledge and their association with the dependent variable. The included covariates were age (years), comorbidity (number of comorbid conditions, Charlson Comorbidity Index), family history of breast cancer (yes, no), disease stage (I, II, III), menopausal status (pre-menopausal, peri/post-menopausal), dietary intake (total daily food energy intake in kilocalories), study location (Edmonton, Calgary), smoking (never, ever), reconstruction surgery at baseline (yes, no), treatment status at baseline (before/during chemotherapy, radiotherapy, hormone therapy), other treatment modalities, and baseline value of the outcome. To address missing data, single imputation by correlation structure was performed at each time point independently. Sensitivity analyses were conducted on complete cases to assess the robustness of the main study findings. All analyses were performed using SPSS version 29 (SPSS Inc., Chicago, IL, USA) and all *p*-values are from two-sided tests, values less than 0.05 were considered statistically significant.

## 3. Results

Of 1528 participants at baseline, 1350 (88.4%) had available data for the analysis at the one-year follow-up. Table 1 presents the sociodemographic and clinical characteristics of the study participants, overall (*n* = 1350) and by chemotherapy treatment. Compared with the non-chemotherapy group, women who received chemotherapy were generally younger, had higher income and kilocalorie intake, more likely to present with Stage II, Grade 3 breast cancer, and to and undergo a mastectomy, radiotherapy, and targeted therapy. At the one-year assessment, AMBER participants overall exhibited statistically significant increases in upper and lower body strength (Table S1). Conversely, statistically significant increases in fat mass (total and percentage), and decreases in percentage lean mass, lean-to-fat ratio, bone mineral density, and bone mineral content were observed.

Women who underwent chemotherapy experienced a statistically significant smaller increase in upper body strength (−1.7 kg, 95%CI: −3.0 to −0.5), and a statistically significant greater decline in lower body muscular endurance (−118 kg, 95%CI: −217 to −19) compared with those who did not receive chemotherapy (Table 2). No significant between-group differences in cardiorespiratory fitness were observed. For body composition (Table 3), those who had chemotherapy had a statistically significant smaller increase in body weight (−1.3 kg, 95%CI: −2.2 to −0.4) and body mass index (BMI, −0.4 kg/m^2^, 95%CI: −0.8 to −0.1), and greater decreases in total lean mass (−0.7 kg, 95%CI: −1.1 to −0.3), bone mineral density (−0.01 g/cm^2^, 95%CI: −0.02 to 0.00), and bone mineral content (−0.04 kg, 95%CI: −0.06 to −0.02) compared to those who did not.

**Figure 1:**
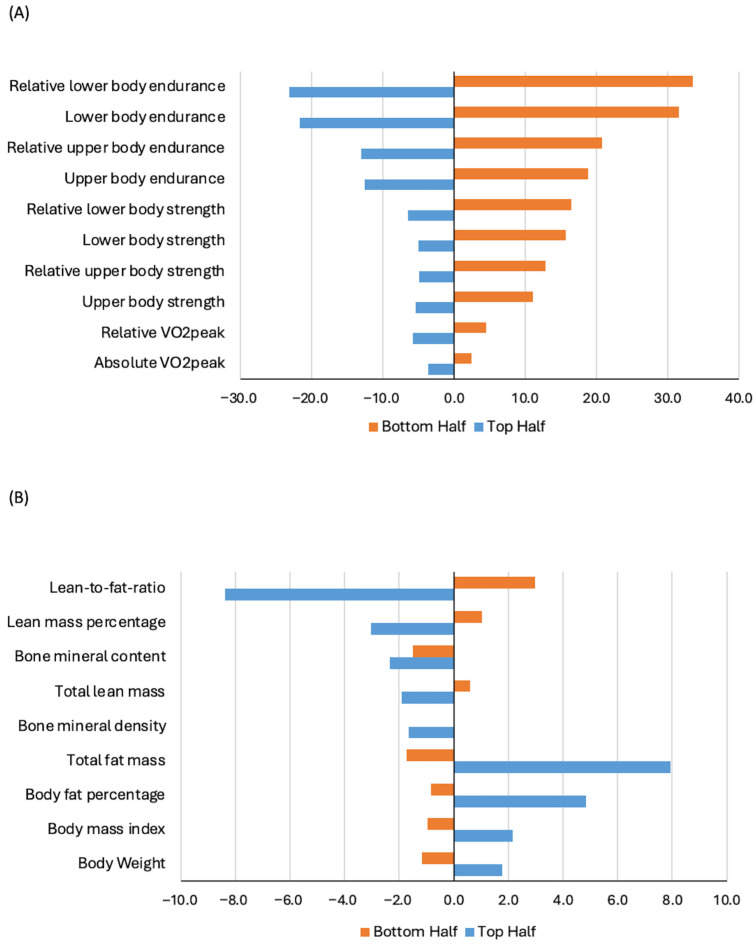
Percentage change in cardiorespiratory and muscular fitness (Panel (**A**)) and body composition (Panel (**B**)) from baseline to one year, stratified by baseline fitness level, among participants treated with chemotherapy in the AMBER cohort study. Note: median split: top vs. bottom half, with the top half representing more favorable baseline fitness.

Stratified analysis within the chemotherapy group showed that baseline fitness level was statistically significantly associated with one-year changes in all HRF components (Table 4 and Table 5). Among women treated with chemotherapy, those with more favorable baseline fitness levels experienced the most pronounced declines across all HRF components (all *p* ≤ 0.001). Figure 1 shows percentage of change from baseline to one year, stratified by median baseline fitness level. When stratified by age, older women (≥60 years) experienced statistically significant greater declines in absolute VO_2peak_, body weight, and BMI compared to younger women receiving chemotherapy (Tables S2 and S3). Stratified analysis by chemotherapy regimen showed that women undergoing anthracycline-based chemotherapy experienced a statistically significant greater decrease in absolute VO_2peak_ and a smaller increase in total fat mass compared with those receiving taxane-based regimens (Tables S4 and S5). Lower RDI (<85%) was associated with statistically significant greater declines in relative VO_2peak_ and upper body endurance, and increases in body weight and BMI (Tables S6 and S7) compared with women who received higher RDI chemotherapy.

No statistically significant differences in any HRF component were found when comparing women who received radiotherapy to those who did not (Tables S8 and S9). Hormone therapy was statistically significantly associated with smaller gains in both absolute and relative lower body strength compared with those who did not receive hormone therapy; however, no significant differences in body composition changes were found between groups (Tables S10 and S11). Targeted therapy was statistically significantly associated with greater declines in relative VO_2peak_ and increases in both absolute and relative lower body endurance; conversely, no statistically significant differences in body composition changes were observed compared with no targeted therapy (Tables S12 and S13). No significant differences in cardiorespiratory or muscular fitness changes were observed between mastectomy and lumpectomy in the neoadjuvant setting, though the mastectomy group showed greater lean mass reduction (Tables S14 and S15). A summary of the statistically significant associations is presented in Table 6 and illustrated in Figure S1. Sensitivity analyses for completed cases were consistent in terms of magnitude and significance of the associations.

**Figure 2:**
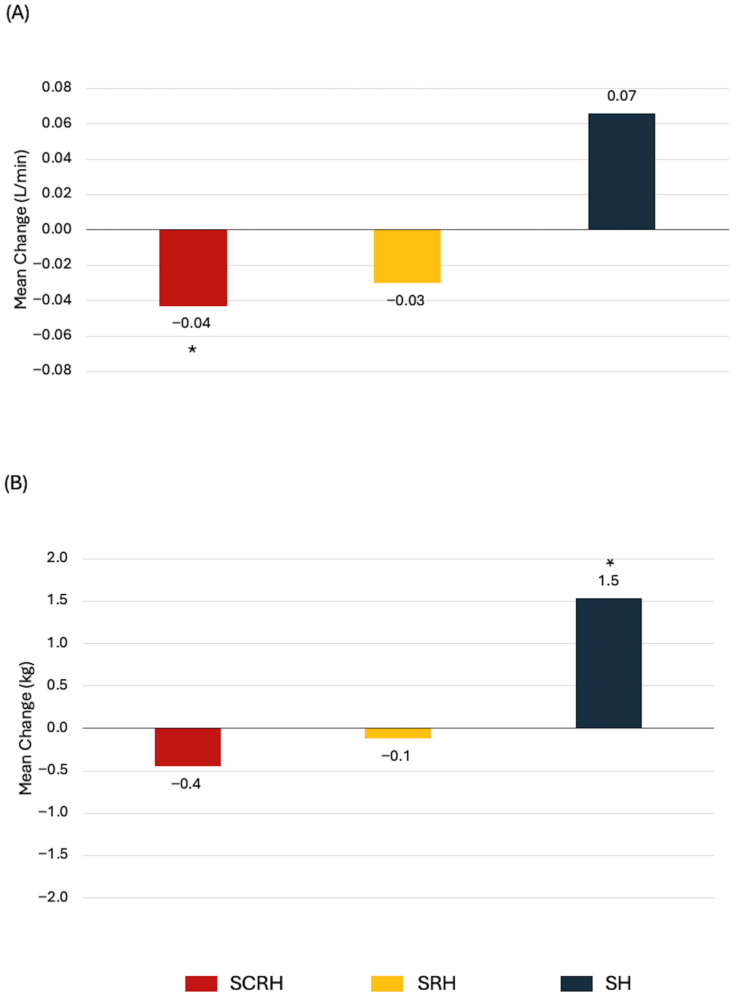

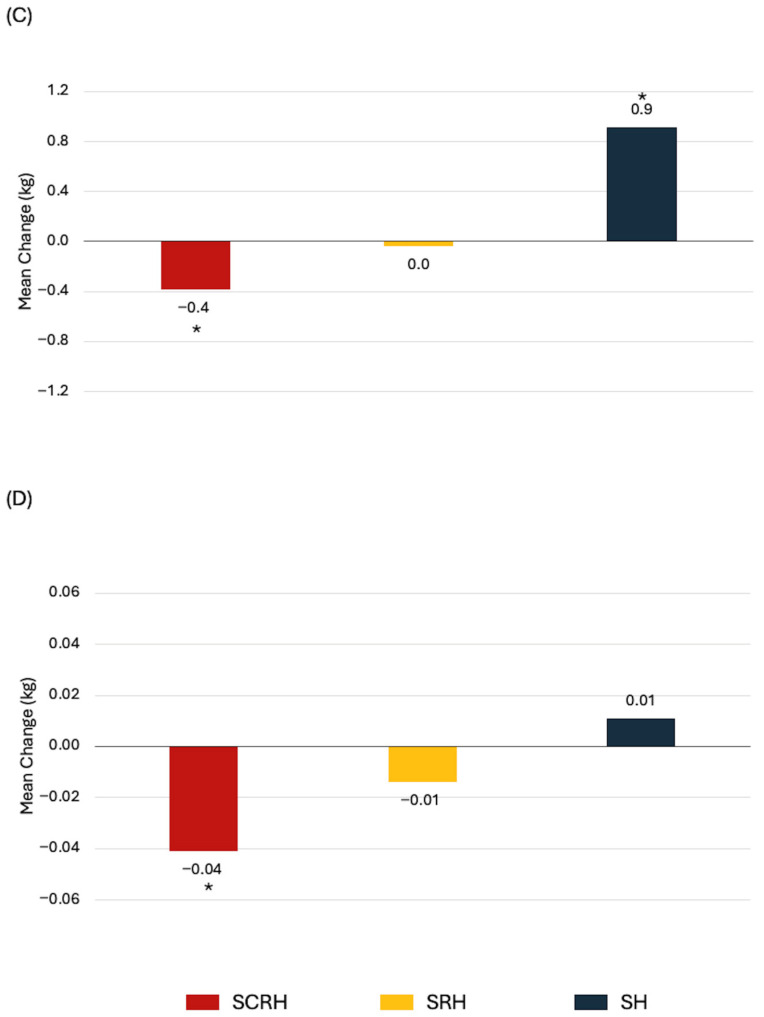
Statistically significant associations between treatment combinations and health-related fitness changes from baseline to 1-year in newly diagnosed breast cancer patients. (**A**) Absolute VO_2peak_, (**B**) Body weight, (**C**) Total lean mass, (**D**) Bone mineral content. * *p* < 0.05 from paired *t*-test.

The most common treatment combinations in our dataset were SCRH (29%), SRH (23%), and SH (11%). Treatment combinations were statistically significantly associated with changes in cardiorespiratory fitness and body composition, but not with muscular strength or endurance (Table S16). Women treated with SCRH experienced the greatest declines in absolute VO_2peak_, total lean mass, and bone mineral content (Figure 2). Exploratory analysis associating the number of therapies (≤2 vs. 3 vs. 4 vs. 5) with changes in HRF yielded no significant findings.

## 4. Discussion

In this large cohort of early-stage breast cancer patients, women who received chemotherapy—either alone or in combination with other treatments—experienced the greatest negative impact on HRF components. Among women treated with chemotherapy, the most pronounced declines were observed in those with more favorable baseline fitness levels. Furthermore, older women and those receiving anthracycline-based chemotherapy experienced greater declines in cardiorespiratory fitness compared with younger women and those treated with taxane-based chemotherapy. Our findings highlight that, while HRF changes differ according to therapeutic approach and patient characteristics, breast cancer patients receiving chemotherapy are particularly affected.

At one-year, participants overall exhibited no changes in cardiorespiratory fitness. Modest improvements were seen in muscle strength; however, this did not translate into clinically meaningful changes. Our findings are potentially explained by the timing of the assessments in relation to treatments. Baseline assessments were conducted after surgery in 90.5% of the participants (38.5% mastectomies and 61.5% lumpectomies), and sometimes after the initiation of chemotherapy (18.0%), radiation therapy (5.6%), or hormone therapy (12.8%). Consequently, participants may already have experienced some declines in HRF components prior to baseline measurement, missing the full negative impact of treatments on cardiorespiratory fitness and muscular fitness. Moreover, at the time of one-year follow-up assessment, many patients would have completed treatments at least several months ago and the acute effects of some treatments (i.e., chemotherapy and radiotherapy) may have dissipated. Despite slight improvements in muscle strength, we observed significant adverse changes in body composition not reflected in body weight, including increases in body fat, along with reductions in lean body mass, and declines in both bone mineral density and bone mineral content. These findings are consistent with previous literature [24,25,26] and are concerning because higher postdiagnosis body fatness increases the risk of all-cause mortality, breast cancer-specific mortality, and second primary breast cancer [27]. Therefore, lifestyle interventions such as exercises aiming to reduce fat mass and maintain or increase lean body mass should be encouraged during and after treatment [28].

We found that breast cancer patients who received chemotherapy experienced a significantly attenuated increase in upper body muscle strength compared with those who did not (1.4% vs. 6.1%, respectively), and a meaningful decrease in lower body endurance (–7.3% vs. 4.4%, respectively) over one year. Our findings are partially consistent with those of Klassen et al., who reported that chemotherapy was associated with reduced strength in both upper and lower body extremities, and greater muscular fatigue compared with no chemotherapy among 255 breast cancer patients [14]. Additionally, we observed a 0.3 kg decrease in lean mass among women who received chemotherapy, consistent with a previous prospective study [13] that reported a 0.4 kg reduction at one-year in breast cancer patients treated with chemotherapy. Their observed lean mass loss occurred primarily in the lower body [13], which may help explain our findings of decreased lower body endurance without significant changes in upper body endurance.

Among women who received chemotherapy, the most pronounced and clinically meaningful declines in HRF components were observed in those with more favorable baseline fitness levels. Several explanations may account for this pattern. One possibility is that individuals with higher initial physiological reserves may experience greater absolute declines over time. Such reductions in physiological reserve could reflect a combination of negative lifestyle changes (e.g., reduced physical activity) and the cumulative adverse effects of anticancer therapies [29]. Additionally, fitter and younger patients might be more likely to receive full-dose standard therapy, given their greater capacity to tolerate aggressive treatment. In contrast, older or less fit patients may require treatment adjustments including systemic treatment de-escalation due to pre-existing comorbidities or concerns about toxicity [30]. Another plausible explanation is regression to the mean, a statistical phenomenon in which higher and lower baseline scores tend to move closer to the group average during follow-up due to random fluctuations in measurement or performance [31].

Nevertheless, chemotherapy itself is associated with skeletal muscle deconditioning in breast cancer patients, characterized as a decrease in both muscle function and mass [32]. While the exact mechanisms underlying chemotherapy-induced skeletal muscle deconditioning are not fully understood, studies involving muscle biopsy analysis following chemotherapy in breast cancer patients have suggested a decrease in the cross-sectional area of muscle fiber, a shift in muscle fiber phenotype (i.e., reduced proportion of type I muscle fibers–primarily responsible for endurance activities), an imbalance between protein synthesis and protein degradation, and alterations in mitochondrial quantity, function, and dynamics [32,33]. Even a single dose of chemotherapy (i.e., epirubicin) can induce rapid and profound skeletal muscle atrophy, reducing type I and type IIa vastus lateralis fiber cross-sectional area by 25% within 4 days, an effect comparable to six decades of healthy aging [34]. Taken together, the physiological changes described in the literature and our findings highlight the importance of exercise interventions that prioritize improving lower body endurance and promoting muscle mass gain as strategies to counteract the detrimental effects of chemotherapy on HRF. Current exercise guidelines for cancer survivors recommend engaging in a minimum of 150 min of moderate-intensity aerobic activity or 75–150 min of vigorous activity weekly, plus strength training on at least two days per week [35].

We also observed a greater decline in absolute VO_2peak_ among older women and those who received anthracycline-based chemotherapy. Older women may be particularly affected because of the confluence of natural and accelerated aging from chemotherapy, which induces DNA damage, overwhelms the repair systems, promotes genomic instability, and accelerates cellular senescence [36]. Moreover, chemotherapy can damage cardiomyocytes leading to cardiotoxicity that impairs cardiorespiratory fitness. Anthracyclines in particular are chemotherapeutic agents known to induce cardiotoxicity, and exposure to these drugs has been associated with higher incidence of left ventricular systolic dysfunction and congestive heart failure [37]. In a cross-sectional study, Koelwyn et al. [38] investigated the integrative effects of the heart and vasculature (i.e., ventricular-arterial coupling) in breast cancer patients, on average of 6.5 years after anthracycline-based chemotherapy. Their findings revealed significant impairments in ventricular-arterial coupling compared to matched controls, primarily due to decreased left ventricular contractility. In a prospective study, Kirkham et al. [39] observed decreases in overall fitness and leg strength during anthracycline- and/or trastuzumab-based chemotherapy for early-stage breast cancer. While these measures of HRF returned to pre-chemotherapy levels after one year, the authors noted that increased fat infiltration into the muscle and changes in muscle energy metabolism persisted. Collectively, these findings suggest that anthracycline-based chemotherapy can have lasting effects on left ventricular contractility and muscle health, potentially contributing to reduced cardiorespiratory fitness.

Other treatment modalities were modestly and inconsistently associated with HRF changes. Targeted therapy was associated with greater declines in relative VO_2peak_, which, in part, may reflect the weight gain observed in patients undergoing targeted therapy and the fact that targeted therapy generally lasts for 12 months. Nevertheless, targeted therapy is often administered in combination with chemotherapy, which can exacerbate cardiotoxicity [37], potentially leading to reduced cardiorespiratory fitness. The association of hormone therapy with impaired gains in lower body strength could be explained by musculoskeletal events (e.g., myalgia, joint stiffness, tingling) in breast cancer patients receiving adjuvant aromatase inhibitors and tamoxifen [40], and the high prevalence of arthralgia during aromatase inhibitor treatment in early breast cancer patients [41,42]. Although hormone therapy has been associated with bone mineral density and bone mineral content loss, we did not find a significant difference compared with patients not receiving hormone therapy. This finding might be because bone loss related to hormone therapy is often more noticeable over the long term [12,26].

We found that treatment combinations uniquely affected cardiorespiratory fitness and body composition but not muscular fitness. Lakoski et al. [16], in a cross-sectional study, examined whether cardiorespiratory fitness differed as a function of adjuvant therapy in 180 early-stage breast cancer patients. Although participants were, on average, seven years after primary adjuvant therapy, cancer survivors who received multimodal treatment (surgery plus chemotherapy and radiotherapy) exhibited the lowest levels in cardiorespiratory fitness compared with surgery-only or single-modality (surgery plus radiation or chemotherapy). In the AMBER study, the number of treatment modalities received was not associated with changes in HRF components; however, specific combinations—especially the one including chemotherapy—did negatively impact HRF.

The modern cancer treatment landscape is characterized by a variety of treatment combinations and sequencing, where the feasibility and effectiveness of exercise interventions, whether as supportive care or as cancer treatment, may differ [43]. In the recent CHALLENGE (Colon Health and Lifelong Exercise Change) trial, a structured exercise program initiated after surgery and adjuvant chemotherapy for colon cancer survivors resulted in sustained improvements in HRF, along with a 28% lower risk of recurrence or new primary cancer and a 37% reduction in all-cause mortality compared to controls [18], highlighting the importance of exercise interventions as a countermeasure to treatment-related declines in fitness and as a strategy to improve long-term clinical outcomes for cancer survivors. It is possible that the structured exercise program tested in the CHALLENGE trial may similarly benefit breast cancer patients who undergo a sequence of surgery followed by chemotherapy. However, further research is necessary to determine the extent to which these benefits translate across cancer types and treatment pathways, and to identify which exercise prescriptions are most effective within distinct clinical scenarios.

Our study has several strengths and limitations. Among prospective cohort studies, the AMBER study has the largest sample of early-stage breast cancer patients that allows us to conduct subgroup analyses of primary treatment modalities. Additionally, we used gold-standard measures to assess a comprehensive set of HRF components. Finally, this study is the first to explore the associations of treatment combinations with changes in distinct HRF components. In terms of limitations, we were unable to conduct HRF assessments before treatment, including surgery, which may have decreased our ability to capture the full impact of treatments on HRF. The one-year follow-up assessment limited our ability to capture any acute effects of the different treatments and combinations. Consistent with the Exercise Across the Postdiagnosis Cancer Continuum (EPiCC) Framework, we recommend that future cohort studies consider the timing of HRF measures in relation to cancer treatment-related time periods (e.g., before any treatments, between treatments, immediately after treatments, during survivorship) [43] to understand the synergistic and cumulative effects of different treatment modalities and combinations on HRF components. Other limitations include small sample sizes for some treatment combinations, which decreased the power to detect significant associations, and the increased risk of Type I error due to multiple comparisons. Despite the healthier participants in our study, findings remain relevant to the broader AMBER-eligible breast cancer population given they often receive similar treatments and combinations.

## 5. Conclusions

Findings from our study revealed that different cancer treatment modalities distinctively impact specific HRF components in early-stage breast cancer. Nevertheless, chemotherapy, either alone or in combination with other treatments, is the most detrimental to body composition and muscular strength and endurance. Moreover, fitter patients appear to experience the greatest negative impact. Future observational studies should examine HRF measures across distinct cancer treatment periods to inform tailored exercise interventions that optimize clinical and patient-reported outcomes, both during and after early-stage breast cancer treatment.

## Figures and Tables

**Table 1:** Sociodemographic and clinical characteristics of the AMBER cohort who completed the 1 year follow-up, overall and by chemotherapy, Alberta, *n* = 1350.

Variables	Overall (*n* = 1350)	No Chemotherapy (*n* = 553)	Chemotherapy (*n* = 797)	*p* Value
Age, years	55.6 ± 10.7	59.3 ± 10.2	52.9 ± 10.3	<0.001
Race, White	1168 (86.5)	495 (89.5)	673 (84.4)	0.007
Married/common-law	1026 (76.0)	416 (75.2)	610 (76.5)	0.58
College/high school or below	717 (53.1)	306 (55.3)	411 (51.6)	0.17
Income ≥ $100,000	706 (52.3)	261 (47.2)	445 (55.8)	0.002
Ever smoker	578 (42.8)	239 (43.2)	339 (42.5)	0.80
Alcohol consumed (g/day)	8.0 ± 18.4	8.6 ± 21.7	7.5 ± 15.7	0.28
Kilocalorie intake (kcal/day)	1727.7 ± 754.3	1666.4 ± 744.4	1770.2 ± 758.6	0.013
Menopausal status				
Premenopausal	357 (26.4)	98 (17.7)	259 (32.5)	<0.001
Postmenopausal	993 (73.6)	455 (82.3)	538 (67.5)	
Weight, kg	73.6 ± 15.6	73.3 ± 15.2	73.8 ± 15.9	0.50
Body mass index, kg/m^2^	27.5 ± 5.6	27.6 ± 5.4	27.4 ± 5.6	0.65
Tumor stage				
Stage I	602 (44.6)	389 (70.3)	213 (26.7)	<0.001
Stage II	626 (46.4)	159 (28.8)	467 (58.6)	
Stage III	122 (9.0)	5 (0.9)	117 (14.7)	
Tumor grade				
Grade 1	165 (12.2)	132 (23.9)	33 (4.1)	<0.001
Grade 2	582 (43.1)	322 (58.2)	260 (32.6)	
Grade 3	603 (44.7)	99 (17.9)	504 (63.2)	
Tumor subtype				
Hormone receptor + HER2 +	170 (12.6)	4 (0.7)	166 (20.8)	<0.001
Hormone receptor + HER2 −	1039 (77.0)	540 (97.6)	499 (62.6)	
Hormone receptor − HER2 −	98 (7.3)	7 (1.3)	91 (11.4)	
Hormone receptor − HER2 +	43 (3.2)	2 (0.4)	41 (5.1)	
Breast cancer surgery				
Lumpectomy	794 (58.8)	377 (68.2)	417 (52.3)	<0.001
Mastectomy	556 (41.2)	176 (31.8)	380 (47.7)	
Chemotherapy	797 (59.0)	-	797 (59.0)	-
Radiotherapy	1009 (74.7)	378 (68.4)	631 (79.2)	<0.001
Hormonal therapy	1111 (82.3)	461 (83.4)	650 (81.6)	0.39
Targeted therapy	217 (16.1)	0.0 (0.0)	217 (27.2)	<0.001
Neoadjuvant chemotherapy	104 (7.7)	0.0 (0.0)	104 (13.0)	<0.001

Continuous variables are reported as Mean (SD). Categorical variables are reported as Counts (% within group).

**Table 2:** Associations of chemotherapy with change in cardiorespiratory and muscular fitness at 1 year in the AMBER cohort study, (*n* = 1350).

			Baseline to One-Year			
Variable	Baseline	One-Year	Within-Group Change	*p* Value	Between-Group Difference ^a^	*p* Value
	Mean ± SD	Mean ± SD	Mean Change (95%CI)		Mean Difference (95%CI)	
Relative VO_2peak_ (mL/kg/min)						
Chemotherapy	26.6 ± 5.8	26.3 ± 5.7	−0.4 (−0.7 to −0.1)	0.020	−0.1 (−0.8 to 0.5)	0.64
No chemotherapy	25.7 ± 5.9	25.7 ± 5.6	0.0 (−0.4 to 0.4)	0.98		
Absolute VO_2peak_ (L/min)						
Chemotherapy	1.91 ± 0.38	1.89 ± 0.38	−0.02 (−0.04 to 0.00)	0.037	−0.04 (−0.09 to 0.00)	0.06
No chemotherapy	1.84 ± 0.39	1.85 ± 0.40	0.01 (−0.02 to 0.04)	0.40		
Upper body strength (kg)						
Chemotherapy	36.6 ± 10.4	37.1 ± 10.2	0.5 (−0.1 to 1.1)	0.13	−1.7 (−3.0 to −0.5)	0.007
No chemotherapy	34.7 ± 9.5	36.8 ± 10.4	2.1 (1.3 to 2.8)	<0.001		
Relative upper body strength (kg/kg)						
Chemotherapy	0.51 ± 0.14	0.51 ± 0.15	0.01 (0.00 to 0.01)	0.15	−0.01 (−0.03 to 0.01)	0.18
No chemotherapy	0.48 ± 0.13	0.51 ± 0.14	0.03 (0.01 to 0.04)	<0.001		
Lower body strength (kg)						
Chemotherapy	99.8 ± 33.0	101.7 ± 32.7	1.9 (−0.1 to 3.9)	0.06	−2.9 (−6.7 to 0.9)	0.14
No chemotherapy	91.3 ± 28.6	96.6 ± 30.0	5.3 (2.9 to 7.6)	<0.001		
Relative lower body strength (kg/kg)						
Chemotherapy	1.38 ± 0.44	1.40 ± 0.44	0.03 (−0.00 to 0.05)	0.07	0.00 (−0.06 to 0.05)	0.90
No chemotherapy	1.27 ± 0.38	1.33 ± 0.41	0.07 (0.04 to 0.10)	<0.001		
Upper body endurance (kg)						
Chemotherapy	499 ± 224	489 ± 219	−11 (−25 to 4)	0.16	−16 (−44 to 11)	0.25
No chemotherapy	462 ± 202	478 ± 199	16 (−1 to 34)	0.07		
Relative upper body endurance (kg/kg)						
Chemotherapy	6.95 ± 3.31	6.81 ± 3.29	−0.15 (−0.36 to 0.07)	0.17	−0.09 (−0.49 to 0.32)	0.68
No chemotherapy	6.46 ± 2.94	6.64 ± 2.89	0.18 (−0.07 to 0.43)	0.15		
Lower body endurance (kg)						
Chemotherapy	1355 ± 849	1257 ± 731	−98 (−158 to −38)	0.001	−118 (−217 to −19)	0.019
No chemotherapy	1142 ± 734	1192 ± 670	50 (−15 to 115)	0.13		
Relative lower body endurance (kg/kg)						
Chemotherapy	18.52 ± 11.16	17.15 ± 9.77	−1.37 (−2.20 to −0.54)	0.001	−0.98 (−2.32 to 0.36)	0.15
No chemotherapy	15.83 ± 10.18	16.31 ± 8.89	0.48 (−0.41 to 1.37)	0.29		

^a^ Adjusted for age, comorbidity, family history of breast cancer, cancer stage, menopausal status, kilocalorie intake, study location, smoking, baseline value of the outcome, treatment status at baseline, other treatment modalities, and reconstruction surgery. Sample sizes: chemotherapy *n* = 797, no chemotherapy *n* = 553.

**Table 3:** Association of chemotherapy with change in body composition at 1 year in the AMBER cohort study, (*n* = 1350).

			Baseline to One-Year			
Variable	Baseline	One-Year	Within-Group Change	*p* Value	Between-Group Difference ^a^	*p* Value
	Mean ± SD	Mean ± SD	Mean change (95%CI)		Mean Difference (95%CI)	
Body weight (kg)						
Chemotherapy	73.8 ± 15.9	73.9 ± 15.7	0.1 (−0.3 to 0.5)	0.76	−1.3 (−2.2 to −0.4)	0.005
No chemotherapy	73.3 ± 15.2	73.8 ± 15.1	0.5 (0.0 to 1.0)	0.05		
Body mass index (kg/m^2^)						
Chemotherapy	27.4 ± 5.6	27.5 ± 5.6	0.1 (0.0 to 0.3)	0.18	−0.4 (−0.8 to −0.1)	0.021
No chemotherapy	27.6 ± 5.4	27.7 ± 5.5	0.1 (−0.1 to 0.3)	0.34		
Total lean mass (kg)						
Chemotherapy	37.9 ± 5.4	37.6 ± 5.3	−0.3 (−0.5 to −0.1)	0.001	−0.7 (−1.1 to −0.3)	<0.001
No chemotherapy	37.4 ± 5.3	37.7 ± 5.4	0.3 (0.1 to 0.6)	0.004		
Total fat mass (kg)						
Chemotherapy	31.5 ± 11.5	32.1 ± 11.4	0.6 (0.2 to 1.0)	0.005	−0.4 (−1.3 to 0.4)	0.30
No chemotherapy	31.7 ± 11.0	32.3 ± 11.2	0.6 (0.2 to 1.1)	0.007		
Lean mass percentage (%)						
Chemotherapy	54.0 ± 6.8	53.5 ± 6.5	−0.7 (−1.0 to −0.4)	<0.001	−0.3 (−0.9 to 0.3)	0.30
No chemotherapy	53.4 ± 6.7	53.2 ± 6.8	−0.2 (−0.5 to 0.2)	0.30		
Body fat percentage (%)						
Chemotherapy	42.8 ± 7.1	43.5 ± 6.9	0.7 (0.4 to 1.0)	<0.001	0.3 (−0.4 to 0.9)	0.39
No chemotherapy	43.3 ± 7.2	43.6 ± 7.3	0.3 (−0.1 to 0.6)	0.13		
Lean-to-fat ratio						
Chemotherapy	1.34 ± 0.46	1.29 ± 0.41	−0.05 (−0.07 to −0.03)	<0.001	−0.02 (−0.06 to 0.02)	0.44
No chemotherapy	1.30 ± 0.43	1.29 ± 0.43	−0.01 (−0.03 to 0.01)	0.29		
Bone mineral density (g/cm^2^)						
Chemotherapy	1.13 ± 0.12	1.12 ± 0.12	−0.01 (−0.02 to −0.01)	<0.001	−0.01 (−0.02 to 0.00)	0.002
No chemotherapy	1.11 ± 0.12	1.11 ± 0.12	0.00 (0.00 to 0.01)	0.55		
Bone mineral content (kg)						
Chemotherapy	2.28 ± 0.36	2.23 ± 0.35	−0.05 (−0.06 to −0.04)	<0.001	−0.04 (−0.06 to −0.02)	<0.001
No chemotherapy	2.21 ± 0.37	2.21 ± 0.37	0.00 (−0.01 to 0.01)	0.88		

^a^ Adjusted for age, comorbidity, family history of breast cancer, cancer stage, menopausal status, kilocalorie intake, study location, smoking, baseline value of the outcome, treatment status at baseline, other treatment modalities, and reconstruction surgery. Sample sizes: chemotherapy *n* = 797, no chemotherapy *n* = 553.

**Table 4:** Associations of baseline fitness levels with changes in cardiorespiratory and muscular fitness at 1 year among the participants treated with chemotherapy in the AMBER cohort study (*n* = 797).

			Baseline to One-Year			
Variable	Baseline	One-Year	Within-Group Change	*p* Value	Between-Group Difference ^a^	*p* Value
	Mean ± SD	Mean ± SD	Mean Change (95%CI)		Mean Difference (95%CI)	
Relative VO_2peak_ (mL/kg/min)						
Top half	31.3 ± 4.3	29.5 ± 5.3	−1.8 (−2.2 to −1.3)	<0.001	−3.2 (−3.8 to −2.6)	<0.001
Bottom half	22.1 ± 2.5	23.1 ± 4.0	1.0 (0.7 to 1.4)	<0.001		
Absolute VO_2peak_ (L/min)						
Top half	2.21 ± 0.27	2.13 ± 0.32	−0.09 (−0.12 to −0.06)	<0.001	−0.16 (−0.20 to −0.12)	<0.001
Bottom half	1.61 ± 0.18	1.65 ± 0.28	0.04 (0.02 to 0.07)	<0.001		
Upper body strength (kg)						
Top half	44.9 ± 7.8	42.5 ± 9.4	−2.4 (−3.3 to −1.5)	<0.001	−5.8 (−7.0 to −4.5)	<0.001
Bottom half	28.9 ± 5.3	32.1 ± 8.2	3.2 (2.4 to 4.0)	<0.001		
Relative upper body strength (kg/kg)						
Top half	0.61 ± 0.11	0.58 ± 0.14	−0.03 (−0.04 to −0.02)	<0.001	−0.08 (−0.09 to −0.06)	<0.001
Bottom half	0.39 ± 0.07	0.44 ± 0.11	0.05 (0.03 to 0.06)	<0.001		
Lower body strength (kg)						
Top half	122.2 ± 27.3	116.1 ± 31.2	−6.1 (−8.9 to −3.4)	<0.001	−19.4 (−23.2 to −15.6)	<0.001
Bottom half	73.3 ± 14.1	84.8 ± 25.6	11.5 (8.9 to 14.1)	<0.001		
Relative lower body strength (kg/kg)						
Top half	1.71 ± 0.34	1.60 ± 0.41	−0.11 (−0.15 to −0.07)	<0.001	−0.29 (−0.34 to −0.24)	<0.001
Bottom half	1.03 ± 0.20	1.20 ± 0.38	0.17 (0.13 to 0.20)	<0.001		
Upper body endurance (kg)						
Top half	661 ± 199	578 ± 230	−83 (−105 to −61)	<0.001	−150 (−178 to −122)	<0.001
Bottom half	335 ± 81	398 ± 165	63 (47 to 80)	<0.001		
Relative upper body endurance (kg/kg)						
Top half	9.25 ± 3.11	8.05 ± 3.58	−1.20 (−1.52 to −0.88)	<0.001	−2.19 (−2.60 to 1.78)	<0.001
Bottom half	4.57 ± 1.09	5.52 ± 2.34	0.95 (0.72 to 1.17)	<0.001		
Lower body endurance (kg)						
Top half	1911 ± 834	1497 ± 809	−414 (−509 to −319)	<0.001	−651 (−764 to −537)	<0.001
Bottom half	760 ± 253	1000 ± 529	240 (187 to 293)	<0.001		
Relative lower body endurance (kg/kg)						
Top half	25.81 ± 10.90	19.84 ± 10.64	−5.97 (−7.24 to −4.70)	<0.001	−9.52 (−11.10 to −7.95)	<0.001
Bottom half	10.67 ± 3.43	14.25 ± 7.77	3.58 (2.77 to 4.38)	<0.001		

Note: median split with the top half representing more favorable baseline fitness. ^a^ Adjusted for age, comorbidity, family history of breast cancer, cancer stage, menopausal status, kilocalorie intake, study location, smoking, treatment status at baseline, other treatment modalities, and reconstruction surgery.

**Table 5:** Associations of baseline fitness levels with changes in body composition at 1 year among the participants treated with chemotherapy in the AMBER cohort study (*n* = 797).

			Baseline to One-Year			
Variable	Baseline	One-Year	Within-Group Change	*p* Value	Between-Group Difference ^a^	*p* Value
	Mean ± SD	Mean ± SD	Mean Change (95%CI)		Mean Difference (95%CI)	
Body weight (kg)						
Top half	61.7 ± 6.1	62.8 ± 7.1	1.1 (0.7 to 1.5)	<0.001	1.9 (1.1 to 2.7)	<0.001
Bottom half	86.0 ± 13.3	85.0 ± 14.0	−1.0 (−1.7 to −0.3)	0.005		
Body mass index (kg/m^2^)						
Top half	23.1 ± 2.1	23.6 ± 2.7	0.5 (0.3 to 0.7)	<0.001	0.8 (0.5 to 1.1)	<0.001
Bottom half	31.7 ± 4.7	31.4 ± 5.0	−0.3 (−0.5 to −0.1)	0.006		
Total lean mass (kg)						
Top half	42.1 ± 4.1	41.3 ± 4.6	−0.8 (−1.1 to −0.5)	<0.001	−1.0 (−1.4 to −0.7)	<0.001
Bottom half	33.8 ± 2.6	34.0 ± 3.0	0.2 (0.0 to 0.4)	0.015		
Total fat mass (kg)						
Top half	22.7 ± 4.7	24.5 ± 5.9	1.8 (1.4 to 2.3)	<0.001	2.1 (1.3 to 2.9)	<0.001
Bottom half	40.3 ± 9.2	39.6 ± 10.5	−0.7 (−1.3 to 0.0)	0.034		
Lean mass percentage (%)						
Top half	59.4 ± 4.7	57.6 ± 5.2	−1.8 (−2.3 to −1.3)	<0.001	−1.9 (−2.5 to 1.3)	<0.001
Bottom half	48.5 ± 3.3	49.0 ± 4.6	0.5 (0.1 to 0.8)	0.019		
Body fat percentage (%)						
Top half	37.1 ± 4.8	38.9 ± 5.4	1.8 (1.3 to 2.2)	<0.001	1.7 (1.1 to 2.4)	<0.001
Bottom half	48.5 ± 3.4	48.1 ± 5.0	−0.4 (−0.8 to 0.0)	0.044		
Lean-to-fat ratio						
Top half	1.67 ± 0.44	1.53 ± 0.40	−0.13 (−0.17 to −0.09)	<0.001	−0.10 (−0.14 to −0.06)	<0.001
Bottom half	1.01 ± 0.14	1.04 ± 0.23	0.03 (0.01 to 0.05)	0.002		
Bone mineral density (g/cm^2^)						
Top half	1.22 ± 0.07	1.20 ± 0.08	−0.02 (−0.03 to −0.02)	<0.001	−0.02 (−0.03 to −0.01)	<0.001
Bottom half	1.04 ± 0.07	1.04 ± 0.07	0.00 (−0.01 to 0.00)	0.023		
Bone mineral content (kg)						
Top half	2.56 ± 0.24	2.50 ± 0.26	−0.06 (−0.08 to −0.05)	<0.001	−0.04 (−0.06 to −0.01)	0.001
Bottom half	1.99 ± 0.18	1.96 ± 0.19	−0.03 (−0.04 to −0.02)	<0.001		

Note: median split with the top half representing more favorable baseline fitness. ^a^ Adjusted for age, comorbidity, family history of breast cancer, cancer stage, menopausal status, kilocalorie intake, study location, smoking, treatment status at baseline, other treatment modalities, and reconstruction surgery.

**Table 6:** Summary of the statistically significant associations between cancer treatment modalities and changes in health-related fitness components at 1 year in the AMBER cohort study. Arrows denote direction of change; ↓ indicates a decrease, ↑ indicates an increase.

	Between-Group Differences in Health-Related Fitness Components			
Treatment Type	Aerobic Fitness	Muscle Strength	Muscle Endurance	Body Composition
**Chemotherapy vs.** **No chemotherapy**	-	↓ Upper body strength (absolute)	↓ Lower body endurance (absolute)	↓ Body weight ↓ Body mass index ↓ Total lean mass ↓ Bone mineral density ↓ Bone mineral content
≥60 years vs. <60 years	↓ Absolute VO_2peak_	-	-	↓ Body weight ↓ Body mass index
Top half vs. Bottom half baseline fitness values	↓ Absolute VO_2peak_ ↓ Relative VO_2peak_	↓ Upper body strength (absolute and relative) ↓ Lower body strength (absolute and relative)	↓ Upper body endurance (absolute and relative) ↓ Lower body endurance (absolute and relative)	↑ Body weight ↑ Body mass index ↓ Total lean mass and lean mass percentage ↑ Total fat mass and body fat percentage ↑ Lean-to-fat ratio ↓ Bone mineral density ↓ Bone mineral content
Anthracycline-based vs. Taxane-based	↓ Absolute VO_2peak_	-	-	↓ Fat mass
RDI < 85% vs. RDI ≥ 85%	↓ Relative VO_2peak_	-	↓ Upper body endurance (relative)	↑ Body weight ↑ Body mass index
**Mastectomy vs. Lumpectomy**	-	-	-	↓ Total lean mass
**Radiotherapy vs.** **No radiotherapy**	-	-	-	-
**Hormone therapy vs.** **No hormone therapy**	-	↓ Lower body strength (absolute and relative)	-	-
**Targeted therapy vs.** **No targeted therapy**	↓ Relative VO_2peak_	-	↑ Lower body endurance (absolute and relative)	-

## Data Availability

The data underlying this article will be shared on reasonable request to the corresponding author.

## Supplementary Materials

The following supporting information can be downloaded at https://www.mdpi.com/article/10.3390/cancers17244026/s1: Table S1. Health-related fitness changes from baseline to one-year in the AMBER cohort study (*n* = 1350); Table S2. Association of age with change in cardiorespiratory and muscular fitness at 1 year among the participants treated with chemotherapy in the AMBER cohort study; Table S3. Association of age with change in body composition at 1 year among the participants treated with chemotherapy in the AMBER cohort study; Table S4. Association of chemotherapy regimen with change in cardiorespiratory and muscular fitness at 1 year in the AMBER cohort study; Table S5. Association of chemotherapy regimen with change in body composition at 1 year in the AMBER cohort study; Table S6. Association of chemotherapy relative dose intensity with change in cardiorespiratory and muscular fitness at 1 year in the AMBER cohort study; Table S7. Association of chemotherapy relative dose intensity with change in body composition at 1 year in the AMBER cohort study; Table S8. Association of radiotherapy with change in cardiorespiratory and muscular fitness at 1 year in the AMBER cohort study; Table S9. Association of radiotherapy with change in body composition at 1 year in the AMBER cohort study; Table S10. Association of hormone therapy with change in cardiorespiratory and muscular fitness at 1 year in the AMBER cohort study; Table S11. Association of hormone therapy with change in body composition at 1 year in the AMBER cohort study; Table S12. Association of targeted therapy with change in cardiorespiratory and muscular fitness at 1 year in the AMBER cohort study; Table S13. Association of targeted therapy with change in body composition at 1 year in the AMBER cohort study; Table S14. Association of breast cancer surgery type with change in cardiorespiratory and muscular fitness at 1 year in patients in the AMBER cohort study who received neoadjuvant therapy; Table S15. Association of breast cancer surgery type with change in body composition at 1 year in patients in the AMBER cohort study who received neoadjuvant therapy; Table S16. Association of breast cancer surgery type with change in body composition at 1 year in patients in the AMBER cohort study who received neoadjuvant therapy; Figure S1. Associations between the individual breast cancer treatment modalities and health-related fitness changes from baseline to 1-year in newly diagnosed breast cancer patients.

## Abbreviations

The following abbreviations are used in this manuscript:

ACSM	American College of Sports Medicine
AMBER	Alberta Moving Beyond Breast Cancer
ANCOVA	Analysis of Covariance
BMI	Body Mass Index
CI	Confidence Interval
EPiCC	Exercise Across the Postdiagnosis Cancer Continuum
HREBA.CC	Health Research Ethics Board of Alberta—Cancer Committee
HRF	Health-related Fitness
RDI	Relative Dose Intensity
SCRH	Surgery, Chemotherapy, Radiotherapy and Hormone Therapy
SH	Surgery and Hormone Therapy
SRH	Surgery, Radiotherapy, and Hormone Therapy
