# Supplementary material for: Associations of Breast Cancer Treatments with One-Year Changes in Health-Related Fitness
Source: Cancers (Basel). 2025 Dec 17;17(24):4026. doi: 10.3390/cancers17244026 (PMC12730790; doi:10.3390/cancers17244026)
Supplement: Supplementary file 1 [file cancers-17-04026-s001.zip › cancers-4015071-supplementary.pdf]

Supplementary Materials

Supplementary Methods
Table S1. Health-related fitness changes from baseline to one-year in the AMBER cohort study (N=1350).
Table S2. Association of age with change in cardiorespiratory and muscular fitness at 1 year among the participants treated with chemotherapy in the AMBER cohort study.
Table S3. Association of age with change in body composition at 1 year among the participants treated with chemotherapy in the AMBER cohort study .
Table S4. Association of chemotherapy regimen with change in cardiorespiratory and muscular fitness at 1 year in the AMBER cohort study.
Table S5. Association of chemotherapy regimen with change in body composition at 1 year in the AMBER cohort study.
Table S6. Association of chemotherapy relative dose intensity with change in cardiorespiratory and muscular fitness at 1 year in the AMBER cohort study.
Table S7. Association of chemotherapy relative dose intensity with change in body composition at 1 year in the AMBER cohort study.
Table S8. Association of radiotherapy with change in cardiorespiratory and muscular fitness at 1 year in the AMBER cohort study.
Table S9. Association of radiotherapy with change in body composition at 1 year in the AMBER cohort study.
Table S10. Association of hormone therapy with change in cardiorespiratory and muscular fitness at 1 year in the AMBER cohort study.
Table S11. Association of hormone therapy with change in body composition at 1 year in the AMBER cohort study.
Table S12. Association of targeted therapy with change in cardiorespiratory and muscular fitness at 1 year in the AMBER cohort study.
Table S13. Association of targeted therapy with change in body composition at 1 year in the AMBER cohort study.
Table S14. Association of breast cancer surgery type with change in cardiorespiratory and muscular fitness at 1 year in patients in the AMBER cohort study who received neoadjuvant therapy.
Table S15. Association of breast cancer surgery type with change in body composition at 1 year in patients in the AMBER cohort study who received neoadjuvant therapy.
Table S16. Association of breast cancer surgery type with change in body composition at 1 year in patients in the AMBER cohort study who received neoadjuvant therapy.
Figure S1. Associations between the individual breast cancer treatment modalities and health-related fitness changes from baseline to 1-year in newly diagnosed breast cancer patients

This supplementary material has been provided by the authors to give readers additional information about their work

Supplementary Methods

Timing of assessments

The goal was to complete the baseline assessment within 90 days of diagnosis and prior to any neoadjuvant/adjuvant therapy, however, patients were allowed to complete baseline assessments before the third cycle of chemotherapy or the tenth fraction of radiation therapy. Patients receiving adjuvant therapy were tested after they recovered from surgery, which was usually 4-6 weeks. Of the 1350 participants, 1222 (90.5%) already had surgery and 318 (23.6%) had started chemotherapy or radiotherapy at the time of the baseline HRF assessments.

Table S1. Health-related fitness changes from baseline to one-year in the AMBER cohort study (N=1350).

	Baseline Mean ± SD	One-year Mean ± SD	Within-group change Mean change (95%CI)	P value
Aerobic fitness				
Relative VO_{2peak} (mL/kg/min)	26.2±5.8	26.0±5.6	-0.2 (-0.5 to 0.0)	.10
Absolute VO_{2peak} (L/min)	1.88±0.39	1.87±0.39	-0.01 (-0.02 to 0.01)	.39
Muscular strength				
Upper body strength (kg)	35.8±10.1	37.0±10.3	1.1 (0.7 to 1.6)	<.001
Relative upper body strength (kg/kg)	0.50±0.14	0.51±0.15	0.01 (0.01 to 0.02)	<.001
Lower body strength (kg)	96.3±31.6	99.6±31.7	3.3 (1.8 to 4.8)	<.001
Relative lower body strength (kg/kg)	1.33±0.42	1.37±0.43	0.04 (0.02 to 0.06)	<.001
Muscular endurance				
Upper body endurance (kg)	484±216	484±21	0 (-11 to 12)	.94
Relative upper body endurance (kg/kg)	6.75±3.17	6.74±3.13	-0.01 (-0.17 to 0.15)	.89
Lower body endurance (kg)	1268±811	1230±707	-38 (-82 to 7)	.10
Relative lower body endurance (kg/kg)	17.42±10.85	16.80±9.42	-0.61 (-1.22 to 0.00)	.05
Body Composition				
Body weight (kg)	73.6±15.6	73.9±15.4	0.3 (-0.1 to 0.6)	.13
Body mass index (kg/m^2)	27.5±5.6	27.6±5.5	0.1 (0.0 to 0.2)	.10
Total lean mass (kg)	37.7±5.4	37.7±5.4	0.0 (-0.2 to 0.1)	.57
Total fat mass (kg)	31.6±11.3	32.2±11.3	0.6 (0.3 to 0.9)	<.001
Lean mass percentage (%)	53.7±6.7	53.3±6.6	-0.5 (-0.7 to –0.2)	<.001
Body fat percentage (%)	43.0±7.1	43.5±7.1	0.5 (0.3 to 0.8)	<.001
Lean-to-fat ratio	1.32±0.45	1.29±0.42	-0.03 (-0.05 to -0.02)	<.001
Bone mineral density (g/cm^2)	1.12±0.12	1.12±0.12	-0.01 (-0.01 to 0.00)	<.001
Bone mineral content (kg)	2.25±0.37	2.22±0.36	-0.03 (-0.04 to -0.02)	<.001

Table S2. Association of age with change in cardiorespiratory and muscular fitness at 1 year among the participants treated with chemotherapy in the AMBER cohort study (N=797)

Variable	Baseline	One-year	Baseline to one-year			
			Within-group change		Between-group difference[a]	
	Mean ± SD	Mean ± SD	Mean change (95%CI)	P	Mean difference (95%CI)	P
Relative VO_{2peak} (mL/kg/min)						
≥60 years	23.8±4.5	23.7±4.7	-0.1 (-0.6 to 0.5)	.80	-0.5 (-1.2 to 0.2)	.14
<60 years	27.7±5.9	27.2±5.7	-0.5 (-0.8 to -0.1)	.012		
Absolute VO_{2peak} (L/min)						
≥60 years	1.71±0.32	1.68±0.34	-0.03 (-0.07 to 0.01)	.12	-0.07 (-0.12 to -0.02)	.004
<60 years	1.99±0.38	1.97±0.37	-0.02 (-0.04 to 0.01)	.13		
Upper body strength (kg)						
≥60 years	32.2±8.2	33.3±8.8	1.0 (0.0 to 2.1)	.054	-1.2 (-2.6 to 0.3)	.12
<60 years	38.2±10.6	38.5±10.3	0.3 (-0.5 to 1.0)	.47		
Relative upper body strength (kg/kg)						
≥60 years	0.45±0.13	0.47±0.14	0.02 (0.01 to 0.04)	.001	0.00 (-0.02 to 0.02)	.79
<60 years	0.53±0.14	0.53±0.14	0.00 (-0.01 to 0.01)	.99		
Lower body strength (kg)						
≥60 years	87.5±25.5	90.1±27.0	2.6 (-1.2 to 6.4)	.18	-3.5 (-8.0 to 1.1)	.14
<60 years	104.3±34.3	106.0±33.6	1.7 (-0.6 to 4.1)	.15		
Relative lower body strength (kg/kg)						
≥60 years	1.22±0.40	1.27±0.40	0.05 (0.00 to 0.11)	.054	-0.03 (-0.09 to 0.04)	.43
<60 years	1.43±0.43	1.45±0.45	0.02 (-0.02 to 0.05)	.34		
Upper body endurance (kg)						
≥60 years	425±174	426±180	1 (-25 to 27)	.95	-29 (-62 to 4)	.09
<60 years	526±233	512±228	-15 (-32 to 3)	.10		
Relative upper body endurance (kg/kg)						
≥60 years	5.96±2.77	6.08±2.94	0.12 (-0.26 to 0.51)	.53	-0.19 (-0.67 to 0.30)	.45
<60 years	7.32±3.42	7.07±3.37	-0.24 (-0.50 to 0.01)	.06		
Lower body endurance (kg)						
≥60 years	1158±695	1135±648	-23 (-121 to 75)	.64	-29 (-147 to 90)	.63
<60 years	1427±888	1301±755	-125 (-199 to -52)	<.001		
Relative lower body endurance (kg/kg)						
≥60 years	15.88±9.11	15.96±9.74	0.09 (-1.38 to 1.56)	.91	0.25 (-1.39 to 1.88)	.77
<60 years	19.47±11.67	17.58±9.75	-1.89 (-2.89 to -0.90)	<.001		

[a]Adjusted for comorbidity, family history of breast cancer, cancer stage, menopausal status, kilocalorie intake, study location, smoking, baseline value of the outcome, treatment status at baseline, other treatment modalities, and reconstruction surgery. Sample sizes: ≥60 years n=212, < 60 years n=585

Table S3. Association of age with change in body composition at 1 year among the participants treated with chemotherapy in the AMBER cohort study (N=797)

Variable	Baseline	One-year	Baseline to one-year			
			Within-group change		Between-group difference[a]	
	Mean ± SD	Mean ± SD	Mean change (95%CI)	P	Mean difference (95%CI)	P
Body weight (kg)						
≥60 years	73.6±14.7	72.3±14.3	-1.3 (-2.0 to -0.6)	<.001	-1.2 (-2.2 to -0.1)	.027
<60 years	73.9±16.4	74.5±16.1	0.6 (0.1 to 1.1)	.024		
Body mass index (kg/m^2)						
≥60 years	27.8±5.3	27.4±5.2	-0.4 (-0.7 to -0.1)	.007	-0.4 (-0.8 to 0.0)	.035
<60 years	27.3±5.7	27.5±5.7	0.3 (0.1 to 0.5)	.002		
Total lean mass (kg)						
≥60 years	36.8±4.8	36.6±4.7	-0.2 (-0.5 to 0.2)	.30	0.1 (-0.3 to 0.6)	.53
<60 years	38.3±5.6	38.0±5.5	-0.3 (-0.5 to -0.1)	.001		
Total fat mass (kg)						
≥60 years	32.6±10.6	31.9±10.6	-0.7 (-1.4 to 0.0)	.07	-0.6 (-1.6 to 0.3)	.20
<60 years	31.1±11.7	32.2±11.7	1.0 (0.6 to 1.5)	<.001		
Lean mass percentage (%)						
≥60 years	52.5±6.6	52.8±6.2	0.3 (-0.3 to 0.9)	.31	0.5 (-0.3 to 1.2)	.20
<60 years	54.5±6.8	53.5±6.6	-1.0 (-1.4 to -0.7)	<.001		
Body fat percentage (%)						
≥60 years	44.5±6.6	44.1±6.5	-0.4 (-0.9 to 0.1)	.13	-0.4 (-1.2 to 0.3)	.27
<60 years	42.1±7.1	43.2±7.1	1.1 (0.7 to 1.5)	<.001		
Lean-to-fat ratio						
≥60 years	1.25±0.46	1.25±0.37	0.00 (-0.05 to 0.05)	.97	0.03 (-0.02 to 0.08)	.19
<60 years	1.37±0.46	1.30±0.43	-0.07 (-0.10 to -0.04)	<.001		
Bone mineral density (g/cm^2)						
≥60 years	1.07±0.11	1.07±0.11	-0.01 (-0.01 to 0.00)	.12	0.00 (-0.01 to 0.01)	.82
<60 years	1.16±0.11	1.14±0.11	-0.02 (-0.02 to -0.01)	<.001		
Bone mineral content (kg)						
≥60 years	2.12±0.34	2.09±0.31	-0.03 (-0.05 to -0.02)	<.001	-0.01 (-0.03 to 0.02)	.61
<60 years	2.33±0.35	2.28±0.35	-0.05 (-0.06 to -0.04)	<.001		

[a]Adjusted for comorbidity, family history of breast cancer, cancer stage, menopausal status, kilocalorie intake, study location, smoking, baseline value of the outcome, treatment status at baseline, other treatment modalities, and reconstruction surgery. Sample sizes: ≥60 years n=212, < 60 years n=585

Table S4. Association of chemotherapy regimen with change in cardiorespiratory and muscular fitness at 1 year in the AMBER cohort study.

Variable	Baseline Mean ± SD	One-year Mean ± SD	Baseline to one-year: Within-group change Mean change (95%CI)	P	Between-group difference[a] Mean difference (95%CI)	P
Relative VO_{2peak} (mL/kg/min)						
Anthracycline-based	26.9 ± 6.0	26.3 ± 5.6	-0.5 (-0.9 to -0.1)	.008	-0.4 (-1.1 to 0.2)	.20
Taxane-based	26.4 ± 5.6	26.2 ± 5.7	-0.2 (-0.7 to 0.2)	.33		
Absolute VO_{2peak} (L/min)						
Anthracycline-based	1.93 ± 0.39	1.89 ± 0.38	-0.04 (-0.07 to -0.01)	.008	-0.06 (-0.10 to -0.01)	.015
Taxane-based	1.91 ± 0.38	1.90 ± 0.38	-0.01 (-0.04 to 0.02)	.61		
Upper body strength (kg)						
Anthracycline-based	36.8 ± 10.7	37.2 ± 10.8	0.4 (-0.5 to 1.4)	.39	0.0 (-1.3 to 1.4)	.97
Taxane-based	36.6 ± 10.13	37.1 ± 9.7	0.5 (-0.3 to 1.4)	.19		
Relative upper body strength (kg/kg)						
Anthracycline-based	0.51 ± 0.15	0.52 ± 0.15	0.01 (-0.01 to 0.02)	.32	0.01 (-0.01 to 0.03)	.36
Taxane-based	0.50 ± 0.14	0.51 ± 0.14	0.01 (-0.01 to 0.02)	.30		
Lower body strength (kg)						
Anthracycline-based	99.5 ± 35.1	101.1 ± 32.9	1.5 (-1.5 to 4.6)	.32	-0.6 (-4.9 to 3.6)	.77
Taxane-based	100.2 ± 31.2	102.4 ± 32.6	2.2 (-0.4 to 4.8)	.10		
Relative lower body strength (kg/kg)						
Anthracycline-based	1.38 ± 0.47	1.41 ± 0.46	0.03 (-0.02 to 0.07)	.25	0.02 (-0.04 to 0.08)	.51
Taxane-based	1.38 ± 0.40	1.40 ± 0.43	0.02 (-0.01 to 0.06)	.19		
Upper body endurance (kg)						
Anthracycline-based	499 ± 231	490 ± 232	-9 (-32 to 15)	.47	13 (-19 to 44)	.43
Taxane-based	500 ± 218	488 ± 209	-12 (-30 to 7)	.23		
Relative upper body endurance (kg/kg)						
Anthracycline-based	6.96 ± 3.32	6.87 ± 3.40	-0.09 (-0.41 to 0.23)	.59	0.31 (-0.15 to 0.77)	.18
Taxane-based	6.95 ± 3.31	6.77 ± 3.20	-0.18 (-0.47 to 0.10)	.20		
Lower body endurance (kg)						
Anthracycline-based	1367 ± 833	1252 ± 756	-115 (-201 to -30)	.008	-10 (-123 to 102)	.86
Taxane-based	1348 ± 864	1264 ± 712	-845 (169 to -1)	.048		
Relative lower body endurance (kg/kg)						
Anthracycline-based	18.83 ± 11.17	17.29 ± 10.40	-1.54 (-2.75 to -0.33)	.013	0.38 (-1.17 to 1.93)	.63
Taxane-based	18.29 ± 11.17	17.06 ± 9.24	-1.23 (-2.38 to -0.09)	.035		

[a]Adjusted for age, comorbidity, family history of breast cancer, cancer stage, menopausal status, kilocalorie intake, study location, smoking, baseline value of the outcome, treatment status at baseline, other treatment modalities, and reconstruction surgery. Sample sizes: Anthracycline-based n=357, Taxane-based n=438

Table S5. Association of chemotherapy regimen with change in body composition at 1 year in the AMBER cohort study.

Variable	Baseline Mean ± SD	One-year Mean ± SD	Baseline to one-year: Within-group change Mean change (95%CI)	P	Between-group difference[a] Mean difference (95%CI)	P
Body weight (kg)						
Anthracycline-based	73.4 ± 15.7	73.3 ± 15.8	-0.2 (-0.7 to 0.4)	.59	-0.7 (-1.7 to 0.2)	.13
Taxane-based	74.2 ± 16.1	74.5 ± 15.7	0.3 (-0.3 to 0.9)	.40		
Body mass index (kg/m^2)						
Anthracycline-based	27.3 ± 5.6	27.3 ± 5.6	0.0 (-0.2 to 0.2)	.75	-0.3 (-0.7 to 0.1)	.12
Taxane-based	27.6 ± 5.7	27.7 ± 5.6	0.2 (-0.1 to -0.4)	.14		
Total lean mass (kg)						
Anthracycline-based	37.7 ± 5.4	37.4 ± 5.5	-0.3 (-0.5 to -0.1)	.013	0.0 (-0.5 to 0.4)	.85
Taxane-based	38.1 ± 5.4	37.8 ± 5.2	-0.3 (-0.1 to 0.0)	.021		
Total fat mass (kg)						
Anthracycline-based	31.3 ± 11.2	31.5 ± 11.3	0.2 (-0.3 to 0.7)	.33	-1.0 (-1.9 to 0.0)	.042
Taxane-based	31.7 ± 11.6	32.6 ± 11.6	0.8 (0.2 to 1.4)	.007		
Lean mass percentage (%)						
Anthracycline-based	54.0 ± 6.6	53.6 ± 6.4	-0.4 (-0.8 to 0.0)	.045	0.5 (-0.2 to 1.2)	.13
Taxane-based	54 ± 6.9	53.1 ± 6.6	-0.9 (-1.4 to -0.4)	<.001		
Body fat percentage (%)						
Anthracycline-based	42.8 ± 7.0	43.2 ± 6.8	0.4 (0.0 to 0.8)	.047	-0.7 (-1.4 to 0.0)	.06
Taxane-based	42.8 ± 7.2	43.7 ± 7.0	0.9 (0.4 to 1.4)	<.001		
Lean-to-fat ratio						
Anthracycline-based	1.33 ± 0.44	1.30 ± 0.42	-0.03 (-0.06 to 0.00)	.030	0.03 (-0.01 to 0.08)	.15
Taxane-based	1.34 ± 0.48	1.28 ± 0.41	-0.07 (-0.10 to -0.03)	<.001		
Bone mineral density (g/cm^2)						
Anthracycline-based	1.14 ± 0.12	1.12 ± 0.12	-0.01 (-0.02 to -0.01)	<.001	0.00 (-0.01 to 0.01)	.63
Taxane-based	1.12 ± 0.11	1.12 ± 0.11	-0.01 (-0.02 to -0.01)	<.001		
Bone mineral content (kg)						
Anthracycline-based	2.27 ± 0.36	2.22 ± 0.36	-0.05 (-0.07 to -0.04)	<.001	0.00 (-0.02 to 0.02)	.88
Taxane-based	2.28 ± 0.35	2.24 ± 0.34	-0.04 (-0.06 to -0.03)	<.001		

[a]Adjusted for age, comorbidity, family history of breast cancer, cancer stage, menopausal status, kilocalorie intake, study location, smoking, baseline value of the outcome, treatment status at baseline, other treatment modalities, and reconstruction surgery. Sample sizes: Anthracycline-based n=357, Taxane-based n=438

Table S6. Association of chemotherapy relative dose intensity with change in cardiorespiratory and muscular fitness at 1 year in the AMBER cohort study.

Variable	Baseline Mean ± SD	One-year Mean ± SD	Baseline to one-year: Within-group change Mean change (95%CI)	P	Between-group difference[a] Mean difference (95%CI)	P
Relative VO_{2peak} (mL/kg/min)						
<85%	25.1 ± 5.7	24.4 ± 5.1	-0.7 (-1.5 to 0.0)	.06	-0.9 (-1.6 to -0.2)	.017
≥85%	27.0 ± 5.8	26.7 ± 5.7	-0.3 (-0.6 to 0.1)	.10		
Absolute VO_{2peak} (L/min)						
<85%	1.86 ± 0.40	1.83 ± 0.35	-0.03 (-0.08 to 0.02)	.21	-0.02 (-0.06 to 0.03)	.52
≥85%	1.93 ± 0.38	1.91 ± 0.39	-0.02 (-0.04 to 0.00)	.09		
Upper body strength (kg)						
<85%	33.6 ± 9.4	35.2 ± 10.2	1.6 (0.2 to 3.1)	.025	0.3 (-1.2 to 1.7)	.69
≥85%	37.4 ± 10.5	37.6 ± 10.2	0.2 (-0.5 to 0.9)	.53		
Relative upper body strength (kg/kg)						
<85%	0.45 ± 0.13	0.47 ± 0.14	0.02 (0.00 to 0.03)	.08	-0.01 (-0.03 to 0.01)	.39
≥85%	0.52 ± 0.14	0.52 ± 0.15	0.00 (-0.01 to 0.01)	.42		
Lower body strength (kg)						
<85%	91.8 ± 29.2	96.7 ± 33.9	4.9 (0.0 to 9.8)	.05	0.1 (-4.5 to 4.7)	.96
≥85%	101.7 ± 33.7	103.0 ± 32.4	1.3 (-0.9 to 3.5)	.25		
Relative lower body strength (kg/kg)						
<85%	1.23 ± 0.41	1.29 ± 0.45	0.05 (-0.01 to 0.12)	.10	-0.03 (-0.10 to 0.03)	.31
≥85%	1.41 ± 0.43	1.43 ± 0.44	0.02 (-0.01 to 0.05)	.22		
Upper body endurance (kg)						
<85%	457 ± 198	438 ± 200	-19 (-50 to 12)	.24	-27 (-61 to 6)	.11
≥85%	509 ± 228	501 ± 222	-8 (-25 to 8)	.32		
Relative upper body endurance (kg/kg)						
<85%	6.15 ± 2.88	5.84 ± 2.82	-0.31 (-0.73 to 0.10)	.14	-0.55 (-1.04 to -0.06)	.029
≥85%	7.14 ± 3.38	7.03 ± 3.35	-0.10 (-0.35 to 0.14)	.40		
Lower body endurance (kg)						
<85%	1291 ± 802	1241 ± 746	-50 (-209 to 109)	.53	-26 (-147 to 95)	.67
≥85%	1370 ± 860	1261 ± 729	-109 (-174 to -45)	.001		
Relative lower body endurance (kg/kg)						
<85%	17.29 ± 11.26	16.07 ± 8.69	-1.21 (-3.37 to 0.95)	.27	-1.28 (-2.94 to 0.38)	.13
≥85%	18.80 ± 11.13	17.40 ± 10.00	-1.40 (-2.30 to -0.50)	.002		

[a]Adjusted for age, comorbidity, family history of breast cancer, cancer stage, menopausal status, kilocalorie intake, study location, smoking, baseline value of the outcome, treatment status at baseline, other treatment modalities, and reconstruction surgery. Sample sizes: RDI<85% n=149, RDI ≥85% n=647.

Table S7. Association of chemotherapy relative dose intensity with change in body composition at 1 year in the AMBER cohort study.

Variable	Baseline	One-year	Baseline to one-year			
			Within-group change		Between-group difference[a]	
	Mean ± SD	Mean ± SD	Mean change (95%CI)	P	Mean difference (95%CI)	P
Body weight (kg)						
<85%	76.6 ± 17.7	77.1 ± 17.4	0.5 (-0.3 to 1.4)	.22	1.2 (0.1 to 2.2)	.028
≥85%	73.2 ± 15.5	73.2 ± 15.2	0.0 (-0.5 to 0.4)	.88		
Body mass index (kg/m^2)						
<85%	28.7 ± 6.2	29.0 ± 6.2	0.2 (-0.1 to 0.6)	.17	0.4 (0.0 to 0.8)	.043
≥85%	27.1 ± 5.4	27.2 ± 5.4	0.1 (-0.1 to 0.2)	.39		
Total lean mass (kg)						
<85%	38.0 ± 5.7	37.7 ± 5.5	-0.3 (-0.7 to 0.1)	.18	0.1 (-0.4 to 0.5)	.70
≥85%	37.8 ± 5.3	37.6 ± 5.3	-0.3 (-0.5 to -0.1)	.003		
Total fat mass (kg)						
<85%	34.1 ± 12.6	34.8 ± 12.7	0.7 (-0.1 to 1.5)	.09	0.8 (-0.3 to 1.8)	.14
≥85%	30.9 ± 11.1	31.5 ± 11.0	0.6 (0.1 to 1.0)	.018		
Lean mass percentage (%)						
<85%	52.1 ± 6.6	51.6 ± 6.3	-0.6 (-1.3 to 0.2)	.13	-0.5 (-1.2 to 0.3)	.23
≥85%	54.4 ± 6.8	53.7 ± 6.5	-0.7 (-1.1 to -0.4)	<.001		
Body fat percentage (%)						
<85%	44.7 ± 6.7	45.3 ± 6.8	0.7 (-0.0 to 1.4)	.05	0.6 (-0.2 to 1.3)	.15
≥85%	42.4 ± 7.1	43.1 ± 6.9	0.7 (0.3 to 1.)	<.001		
Lean-to-fat ratio						
<85%	1.23 ± 0.40	1.19 ± 0.35	-0.04 (-0.10 to 0.02)	.17	-0.03 (-0.08 to 0.02)	.28
≥85%	1.36 ± 0.47	1.31 ± 0.42	-0.05 (-0.08 to -0.03)	<.001		
Bone mineral density (g/cm^2)						
<85%	1.13 ± 0.11	1.12 ± 0.11	-0.02 (-0.02 to -0.01)	.003	0.00 (-0.01 to 0.01)	.65
≥85%	1.13 ± 0.12	1.12 ± 0.12	-0.01 (-0.02 to -0.01)	<.001		
Bone mineral content (kg)						
<85%	2.27 ± 0.33	2.22 ± 0.33	-0.05 (-0.07 to -0.03)	<.001	0.00 (-0.03 to 0.02)	.84
≥85%	2.28 ± 0.36	2.23 ± 0.36	-0.05 (-0.06 to -0.04)	<.001		

[a]Adjusted for age, comorbidity, family history of breast cancer, cancer stage, menopausal status, kilocalorie intake, study location, smoking, baseline value of the outcome, treatment status at baseline, other treatment modalities, and reconstruction surgery. Sample sizes: RDI<85% n=149, RDI≥85% n=647.

Table S8. Association of radiotherapy with change in cardiorespiratory and muscular fitness at 1 year in the AMBER cohort study.

Variable	Baseline	One-year	Baseline to one-year			
			Within-group change		Between-group difference[a]	
	Mean ± SD	Mean ± SD	Mean change (95%CI)	P	Mean difference (95%CI)	P
Relative VO_{2peak} (mL/kg/min)						
Radiotherapy	26.0±5.9	25.8±5.5	-0.3 (-0.6 to 0.0)	.049	0.4 (-1.1 to 0.2)	.21
No radiotherapy	26.8±5.8	26.8±5.9	0.0 (-0.4 to 0.5)	.91		
Absolute VO_{2peak} (L/min)						
Radiotherapy	1.89±0.39	1.87±0.39	-0.02 (-0.04 to 0.00)	.042	-0.02 (-0.07 to 0.02)	.34
No radiotherapy	1.85±0.37	1.88±0.39	0.03 (0.00 to 0.06)	.06		
Upper body strength (kg)						
Radiotherapy	36.0±10.1	36.9±10.3	0.9 (0.3 to 1.4)	.001	-0.7 (-2.0 to 0.7)	.33
No radiotherapy	35.3±9.8	37.1±10.3	1.8 (0.8 to 2.8)	<.001		
Relative upper body strength (kg/kg)						
Radiotherapy	0.49±0.14	0.51±0.15	0.01 (0.01 to 0.02)	.001	-0.01 (-0.03 to 0.01)	.30
No radiotherapy	0.51±0.15	0.53±0.14	0.02 (0.00 to 0.03)	.019		
Lower body strength (kg)						
Radiotherapy	97.3±32.3	100.4±32.4	3.0 (1.3 to 4.8)	.001	-0.5 (-4.6 to 3.7)	.82
No radiotherapy	93.3±29.1	97.4±29.5	4.2 (1.1 to 7.2)	.007		
Relative lower body strength (kg/kg)						
Radiotherapy	1.33±0.42	1.37±0.43	0.04 (0.02 to 0.07)	.001	-0.02 (-0.07 to 0.04)	.58
No radiotherapy	1.34±0.40	1.38±0.42	0.04 (0.00 to 0.09)	.05		
Upper body endurance (kg)						
Radiotherapy	487±216	486±215	-1 (-14 to 12)	.84	-16 (-46 to 14)	.29
No radiotherapy	475±213	481±201	6 (-17 to 29)	.62		
Relative upper body endurance (kg/kg)						
Radiotherapy	6.71±3.15	6.71±3.16	0.00 (-0.18 to 0.18)	.99	-0.22 (-0.66 to 0.22)	.32
No radiotherapy	6.89±3.24	6.85±3.05	-0.04 (-0.39 to 0.30)	.81		
Lower body endurance (kg)						
Radiotherapy	1288±813	1247±717	-41 (-92 to 10)	.11	-0.3 (-141 to 73)	.53
No radiotherapy	1208±802	1181±676	-27 (-118 to 63)	.55		
Relative lower body endurance (kg/kg)						
Radiotherapy	17.47±10.71	16.86±9.29	-0.61 (-1.30 to 0.07)	.08	-0.66 (-2.12 to 0.79)	.37
No radiotherapy	17.24±11.25	16.64±9.81	-0.60 (-1.94 to 0.74)	.38		

[a]Adjusted for age, comorbidity, family history of breast cancer, cancer stage, menopausal status, kilocalorie intake, study location, smoking, baseline value of the outcome, treatment status at baseline, other treatment modalities, and reconstruction surgery. Sample sizes: radiotherapy n= 1009, no radiotherapy n=341.

Table S9. Association of radiotherapy with change in body composition at 1 year in the AMBER cohort study.

Variable	Baseline	One-year	Baseline to one-year			
			Within-group change		Between-group difference[a]	
	Mean ± SD	Mean ± SD	Mean change (95%CI)	P	Mean difference (95%CI)	P
Body weight (kg)						
Radiotherapy	74.5±15.8	74.5±15.6	0.0 (-0.4 to 0.4)	.99	-0.1 (-1.0 to 0.9)	.90
No radiotherapy	70.9±14.8	71.9±14.7	1.0 (0.3 to 1.6)	.003		
Body mass index (kg/m^2)						
Radiotherapy	27.8±5.6	27.8±5.6	0.0 (-0.1 to 0.2)	.82	-0.1 (-0.4 to 0.3)	.73
No radiotherapy	26.5±5.2	26.8±5.3	0.4 (0.1 to 0.6)	.005		
Total lean mass (kg)						
Radiotherapy	37.9±5.4	37.8±5.4	-0.1 (-0.3 to 0.0)	.09	-0.1 (-0.5 to 0.3)	.66
No radiotherapy	37.1±5.2	37.3±5.3	0.2 (-0.1 to 0.5)	.12		
Total fat mass (kg)						
Radiotherapy	32.3±11.4	32.7±11.4	0.4 (0.0 to 0.7)	.029	-0.1 (-1.0 to 0.8)	.84
No radiotherapy	29.4±10.7	30.7±11.0	1.3 (0.6 to 1.9)	<.001		
Lean mass percentage (%)						
Radiotherapy	53.3±6.6	53.0±6.5	-0.4 (-0.6 to -0.1)	.005	-0.1 (-0.8 to 0.6)	.73
No radiotherapy	55.0±7.1	54.2±6.9	-0.8 (-1.3 to -0.3)	.002		
Body fat percentage (%)						
Radiotherapy	43.5±7.0	43.9±7.0	0.4 (0.1 to 0.6)	.005	0.0 (-0.7 to 0.7)	.99
No radiotherapy	41.6±7.5	42.5±7.4	0.9 (0.4 to 1.4)	<.001		
Lean-to-fat ratio						
Radiotherapy	1.29±0.43	1.27±0.41	-0.03 (-0.04 to -0.01)	.005	0.00 (-0.04 to 0.05)	.83
No radiotherapy	1.41±0.50	1.35±0.44	-0.06 (-0.10 to -0.02)	.002		
Bone mineral density (g/cm^2)						
Radiotherapy	1.13±0.12	1.12±0.12	-0.01 (-0.01 to -0.01)	<.001	0.00 (-0.01 to 0.00)	.31
No radiotherapy	1.12±0.12	1.12±0.12	0.00 (-0.01 to 0.00)	.39		
Bone mineral content (kg)						
Radiotherapy	2.25±0.36	2.22±0.35	-0.03 (-0.04 to -0.02)	<.001	-0.01 (-0.03 to 0.01)	.41
No radiotherapy	2.23±0.38	2.22±0.37	-0.01 (-0.03 to 0.01)	.28		

[a]Adjusted for age, comorbidity, family history of breast cancer, cancer stage, menopausal status, kilocalorie intake, study location, smoking, baseline value of the outcome, treatment status at baseline, other treatment modalities, and reconstruction surgery. Sample sizes: radiotherapy n= 1009, no radiotherapy n= 341.

Table S10. Association of hormone therapy with change in cardiorespiratory and muscular fitness at 1 year in the AMBER cohort study.

Variable	Baseline	One-year	Baseline to one-year			
			Within-group change		Between-group difference[a]	
	Mean ± SD	Mean ± SD	Mean change (95%CI)	P	Mean difference (95%CI)	P
Relative VO_{2peak} (mL/kg/min)						
Hormone therapy	26.3±5.9	26.0±5.6	-0.3 (-0.5 to 0.0)	.046	-0.3 (-0.9 to 0.3)	.27
No hormone therapy	26.1±5.6	26.2±5.7	0.0 (-0.7 to 0.7)	.90		
Absolute VO_{2peak} (L/min)						
Hormone therapy	1.88±0.38	1.87±0.39	-0.01 (-0.03 to 0.01)	.22	-0.03 (-0.07 to 0.01)	.19
No hormone therapy	1.89±0.43	1.90±0.41	0.01 (-0.04 to 0.06)	.72		
Upper body strength (kg)						
Hormone therapy	35.9±9.9	36.9±10.1	1.0 (0.5 to 1.5)	<.001	-0.5 (-1.6 to 0.7)	.42
No hormone therapy	35.4±10.6	37.1±11.3	1.7 (0.5 to 2.9)	.005		
Relative upper body strength (kg/kg)						
Hormone therapy	0.50±0.14	0.51±0.14	0.01 (0.00 to 0.02)	.002	-0.01 (-0.02 to 0.01)	.43
No hormone therapy	0.48±0.13	0.51±0.16	0.02 (0.01 to 0.04)	.004		
Lower body strength (kg)						
Hormone therapy	96.8±31.2	99.2±30.8	2.4 (0.8 to 4.1)	.004	-4.0 (-7.6 to -0.4)	.027
No hormone therapy	94.2±33.3	101.7±35.7	7.5 (3.7 to 11.2)	<.001		
Relative lower body strength (kg/kg)						
Hormone therapy	1.34±0.41	1.37±0.42	0.03 (0.01 to 0.05)	.010	-0.05 (-0.10 to 0.00)	.035
No hormone therapy	1.29±0.43	1.39±0.49	0.10 (0.05 to 0.15)	<.001		
Upper body endurance (kg)						
Hormone therapy	483±213	480±207	-3 (-15 to 9)	.62	-21 (-47 to 5)	.11
No hormone therapy	487±227	503±230	17 (-13 to 46)	.26		
Relative upper body endurance (kg/kg)						
Hormone therapy	6.77±3.18	6.70±3.06	-0.07 (-0.24 to 0.11)	.44	-0.29 (-0.66 to 0.09)	.13
No hormone therapy	6.69±3.15	6.94±3.43	0.26 (-0.16 to 0.68)	.23		
Lower body endurance (kg)						
Hormone therapy	1278±822	1232±702	-46 (-95 to 3)	.07	0 (-91 to 92)	.99
No hormone therapy	1223±755	1223±735	1 (-104 to 107)	.98		
Relative lower body endurance (kg/kg)						
Hormone therapy	17.58±10.95	16.83±9.27	-0.75 (-1.43 to -0.08)	.029	-0.06 (-1.30 to 1.19)	.93
No hormone therapy	16.65±10.34	16.70±10.13	0.05 (-1.41 to 1.50)	.95		

[a]Adjusted for age, comorbidity, family history of breast cancer, cancer stage, menopausal status, kilocalorie intake, study location, smoking, baseline value of the outcome, treatment status at baseline, other treatment modalities, and reconstruction surgery. Sample sizes: hormone therapy n=1111, no hormone therapy n=239.

Table S11. Association of hormone therapy with change in body composition at 1 year in the AMBER cohort study.

Variable	Baseline	One-year	Baseline to one-year			
			Within-group change		Between-group difference[a]	
	Mean ± SD	Mean ± SD	Mean change (95%CI)	P	Mean difference (95%CI)	P
Body weight (kg)						
Hormone therapy	73.5 ± 15.5	73.7 ± 15.4	0.2 (-0.1 to 0.6)	.20	-0.2 (-1.0 to 0.7)	.66
No hormone therapy	74.1 ± 16.1	74.5 ± 15.4	0.4 (-0.5 to 1.2)	.43		
Body mass index (kg/m^2)						
Hormone therapy	27.5 ± 5.6	27.6 ± 5.5	0.1 (-0.1 to 0.2)	.22	-0.1 (-0.4 to 0.2)	.64
No hormone therapy	27.4 ± 5.5	27.6 ± 5.5	0.2 (-0.1 to 0.5)	.24		
Total lean mass (kg)						
Hormone therapy	37.7 ± 5.3	37.6 ± 5.3	-0.1 (-0.2 to 0.1)	.33	-0.2 (-0.6 to 0.1)	.21
No hormone therapy	37.7 ± 5.8	37.8 ± 5.6	0.1 (-0.3 to 0.5)	.54		
Total fat mass (kg)						
Hormone therapy	31.5 ± 11.3	32.1 ± 11.3	0.7 (0.4 to 1.0)	<.001	0.2 (-0.6 to 1.0)	.64
No hormone therapy	32.1 ± 11.3	32.4 ± 11.3	0.3 (-0.6 to 1.2)	.52		
Lean mass percentage (%)						
Hormone therapy	53.8 ± 6.8	53.3 ± 6.6	-0.6 (-0.8 to -0.3)	<.001	-0.3 (-0.9 to 0.2)	.25
No hormone therapy	53.4 ± 6.5	53.3 ± 6.7	-0.1 (-0.7 to 0.5)	.70		
Body fat percentage (%)						
Hormone therapy	42.9 ± 7.2	43.5 ± 7.1	0.6 (0.4 to 0.9)	<.001	0.3 (-0.2 to 0.9)	.25
No hormone therapy	43.4 ± 7.0	43.5 ± 7.2	0.1 (-0.6 to 0.8)	.81		
Lean-to-fat ratio						
Hormone therapy	1.33 ± 0.45	1.29 ± 0.42	-0.04 (-0.06 to -0.02)	<.001	-0.02 (-0.06 to 0.01)	.22
No hormone therapy	1.30 ± 0.43	1.29 ± 0.43	-0.01 (-0.05 to 0.03)	.70		
Bone mineral density (g/cm^2)						
Hormone therapy	1.13± 0.12	1.12± 0.12	-0.01 (-0.01 to 0.00)	<.001	0.00 (-0.01 to 0.01)	.78
No hormone therapy	1.11± 0.11	1.10± 0.11	-0.01 (-0.01 to 0.00)	.16		
Bone mineral content (kg)						
Hormone therapy	2.25 ± 0.36	2.22 ± 0.36	-0.03 (-0.04 to -0.02)	<.001	0.00 (-0.02 to 0.02)	.91
No hormone therapy	2.24 ± 0.38	2.21 ± 0.37	-0.03 (-0.05 to 0.00)	.025		

[a]Adjusted for age, comorbidity, family history of breast cancer, cancer stage, menopausal status, kilocalorie intake, study location, smoking, baseline value of the outcome, treatment status at baseline, other treatment modalities, and reconstruction surgery. Sample sizes: hormone therapy n=1111, no hormone therapy n=239.

Table S12. Association of targeted therapy with change in cardiorespiratory and muscular fitness at 1 year in the AMBER cohort study.

Variable	Baseline	One-year	Baseline to one-year			
			Within-group change		Between-group difference[a]	
	Mean ± SD	Mean ± SD	Mean change (95%CI)	P	Mean difference (95%CI)	P
Relative VO_{2peak} (mL/kg/min)						
Targeted therapy	26.7 ± 6.3	25.7 ± 5.7	-0.9 (-1.6 to -0.3)	.003	-0.9 (-1.5 to -0.2)	.006
No targeted therapy	26.2 ± 5.7	26.1 ± 5.6	-0.1 (-0.3 to 0.2)	.62		
Absolute VO_{2peak} (L/min)						
Targeted therapy	1.93 ± 0.42	1.88 ± 0.38	-0.05 (-0.09 to -0.01)	.022	-0.04 (-0.08 to 0.01)	.10
No targeted therapy	1.87 ± 0.38	1.87 ± 0.39	0.00 (-0.02 to 0.02)	.95		
Upper body strength (kg)						
Targeted therapy	36.7 ± 10.2	35.7 ± 10.0	0.5 (-0.7 to 1.7)	.42	0.0 (-1.2 to 1.3)	.97
No targeted therapy	37.2 ± 10.7	36.9 ± 10.2	1.3 (0.7 to 1.8)	<.001		
Relative upper body strength (kg/kg)						
Targeted therapy	0.51 ± 0.15	0.51 ± 0.15	0.00 (-0.02 to 0.02)	.94	-0.01 (-0.03 to 0.01)	.36
No targeted therapy	0.50 ± 0.14	0.51 ± 0.14	0.02 (0.01 to 0.02)	<.001		
Lower body strength (kg)						
Targeted therapy	98.8 ± 33.2	102.0 ± 35.2	3.3 (-0.6 to 7.2)	.10	1.6 (-2.3 to 5.5)	.43
No targeted therapy	95.8 ± 31.2	99.2 ± 31.0	3.3 (1.7 to 5.0)	<.001		
Relative lower body strength (kg/kg)						
Targeted therapy	1.35 ± 0.41	1.38 ± 0.46	0.03 (-0.02 to 0.09)	.21	0.00 (-0.06 to 0.05)	.95
No targeted therapy	1.33 ± 0.42	1.37 ± 0.42	0.04 (0.02 to 0.07)	<.001		
Upper body endurance (kg)						
Targeted therapy	499 ± 231	487 ± 222	-13 (-40 to 15)	.37	-5 (-33 to 23)	.73
No targeted therapy	481 ± 212	484 ± 209	3 (-9 to 15)	.64		
Relative upper body endurance (kg/kg)						
Targeted therapy	6.90 ± 3.37	6.72 ± 3.47	-0.18 (-0.56 to 0.21)	.37	-0.11 (-0.52 to 0.31)	.61
No targeted therapy	6.73 ± 3.13	6.74 ± 3.06	0.02 (-0.16 to 0.20)	.83		
Lower body endurance (kg)						
Targeted therapy	1254 ± 732	1341 ± 876	87 (-30 to 204)	.14	178 (77 to 279)	.001
No targeted therapy	1271 ± 825	1209 ± 669	-61 (-109 to -14)	.012		
Relative lower body endurance (kg/kg)						
Targeted therapy	16.75 ± 8.54	17.80 ± 10.89	1.05 (-0.52 to 2.62)	.19	1.78 (0.41 to 3.16)	.011
No targeted therapy	17.54 ± 11.23	16.61 ± 9.11	-0.93 (-1.60 to -0.26)	.006		

[a]Adjusted for age, comorbidity, family history of breast cancer, cancer stage, menopausal status, kilocalorie intake, study location, smoking, baseline value of the outcome, treatment status at baseline, other treatment modalities, and reconstruction surgery. Sample sizes: targeted therapy n=217, no targeted therapy n=1133

Table S13. Association of targeted therapy with change in body composition at 1 year in the AMBER cohort study.

Variable	Baseline	One-year	Baseline to one-year			
			Within-group change		Between-group difference[a]	
	Mean ± SD	Mean ± SD	Mean change (95%CI)	P	Mean difference (95%CI)	P
Body weight (kg)						
Targeted therapy	74.7 ± 18.4	75.4 ± 18.7	0.7 (0.0 to 1.5)	.046	0.90 (-0.03 to 1.83)	.06
No targeted therapy	73.4 ± 15.1	73.6 ± 14.7	0.2 (-0.2 to 0.5)	.39		
Body mass index (kg/m^2)						
Targeted therapy	27.6 ± 6.4	27.9 ± 6.6	0.3 (0.1 to 0.6)	.013	0.3 (-0.1 to 0.6)	.15
No targeted therapy	27.5 ± 5.4	27.5 ± 7.3	0.1 (-0.1 to 0.2)	.39		
Total lean mass (kg)						
Targeted therapy	38.1 ± 6.0	38.0 ± 5.8	-0.1 (-0.4 to 0.2)	.56	0.3 (-0.1 to 0.7)	.21
No targeted therapy	37.6 ± 5.2	37.6 ± 5.3	-0.3 (-0.2 to 0.1)	.70		
Total fat mass (kg)						
Targeted therapy	32.2 ± 13.3	33.1 ± 13.5	0.9 (0.2 to 1.6)	.018	0.5 (-0.4 to 1.4)	.26
No targeted therapy	31.5 ± 10.9	32.0 ± 10.9	0.6 (0.2 to 0.9)	.001		
Lean mass percentage (%)						
Targeted therapy	54 ± 7.3	52.1 ± 7.0	-0.9 (-1.5 to -0.2)	.008	-0.3 (-0.9 to 0.3)	.34
No targeted therapy	53.7 ± 6.6	53.3 ± 6.6	-0.4 (-0.6 to -0.2)	.001		
Body fat percentage (%)						
Targeted therapy	42.9 ± 7.4	43.6 ± 7.5	0.7 (0.1 to 1.3)	.016	0.1 (-0.5 to 0.8)	.74
No targeted therapy	43.0 ± 7.1	43.5 ± 7.0	0.5 (0.2 to 0.7)	<.001		
Lean-to-fat ratio						
Targeted therapy	1.35 ± 0.50	1.28 ± 0.43	-0.07 (-0.12 to -0.01)	.012	0.00 (-0.04 to 0.04)	.93
No targeted therapy	1.32 ± 0.44	1.29 ± 0.42	-0.03 (-0.05 to -0.01)	.001		
Bone mineral density (g/cm^2)						
Targeted therapy	1.13 ± 0.11	1.12 ± 0.11	-0.01 (-0.02 to 0.00)	.003	0.00 (-0.01 to 0.01)	.79
No targeted therapy	1.12 ± 0.12	1.12 ± 0.12	-0.01 (-0.01 to -0.02)	<.001		
Bone mineral content (kg)						
Targeted therapy	2.28 ± 0.35	2.24 ± 0.36	-0.04 (-0.06 to -0.02)	<.001	0.01 (-0.02 to 0.03)	.55
No targeted therapy	2.24 ± 0.37	2.22 ± 0.36	-0.02 (-0.03 to -0.02)	<.001		

[a]Adjusted for age, comorbidity, family history of breast cancer, cancer stage, menopausal status, kilocalorie intake, study location, smoking, baseline value of the outcome, treatment status at baseline, other treatment modalities, and reconstruction surgery. Sample sizes: targeted therapy n=217, no targeted therapy n=1133

Table S14. Association of breast cancer surgery type with change in cardiorespiratory and muscular fitness at 1 year in patients in the AMBER cohort study who received neoadjuvant therapy.

Variable	Baseline	One-year	Baseline to one-year			
			Within-group change		Between-group difference[a]	
	Mean ± SD	Mean ± SD	Mean change (95%CI)	P	Mean difference (95%CI)	P
Relative VO_{2peak} (mL/kg/min)						
Mastectomy	26.8 ± 5.9	26.4 ± 5.8	-0.5 (-1.5 to 0.6)	.38	-0.1 (-2.0 to 1.8)	.92
Lumpectomy	28.3 ± 7.4	27.5 ± 6.7	-0.8 (-2.5 to 0.9)	.33		
Absolute VO_{2peak} (L/min)						
Mastectomy	1.95 ± 0.37	1.90 ± 0.35	-0.05 (-0.12 to 0.03)	.20	-0.06 (-0.20 to 0.07)	.34
Lumpectomy	2.09 ± 0.45	2.03 ± 0.42	-0.06 (-0.19 to 0.06)	.33		
Upper body strength (kg)						
Mastectomy	37.8 ± 9.5	37.6 ± 10.9	-0.2 (-2.5 to 2.1)	.89	1.7 (-2.4 to 5.8)	.41
Lumpectomy	42.3 ± 9.5	38.6 ± 10.4	-3.7 (-6.5 to -1.0)	.010		
Relative upper body strength (kg/kg)						
Mastectomy	0.51 ± 0.13	0.52 ± 0.14	0.00 (-0.03 to 0.03)	.90	0.03 (-0.03 to 0.08)	.29
Lumpectomy	0.57 ± 0.15	0.52 ± 0.17	-0.05 (-0.09 to -0.01)	.020		
Lower body strength (kg)						
Mastectomy	100.9 ± 33.0	103.7 ± 32.6	2.8 (-4.3 to 10.0)	.44	0.3 (-11.9 to 12.6)	.96
Lumpectomy	114.4 ± 37.6	111.4 ± 36.2	-3.0 (-12.3 to 6.3)	.51		
Relative lower body strength (kg/kg)						
Mastectomy	1.37 ± 0.43	1.43 ± 0.45	0.05 (-0.04 to 0.15)	.26	0.02 (-0.15 to 0.19)	.79
Lumpectomy	1.55 ± 0.57	1.51 ± 0.56	-0.04 (-0.18 to 0.10)	.54		
Upper body endurance (kg)						
Mastectomy	516 ± 197	498 ± 190	-27 (-83 to 29)	.34	-36 (-121 to 49)	.40
Lumpectomy	526 ± 223	529 ± 203	3 (-66 to 71)	.94		
Relative upper body endurance (kg/kg)						
Mastectomy	6.93 ± 2.27	6.73 ± 2.51	-0.20 (-0.90 to 0.50)	.57	-0.24 (-1.43 to 0.94)	.68
Lumpectomy	7.17 ± 3.55	7.21 ± 3.29	0.04 (-0.95 to 1.04)	.93		
Lower body endurance (kg)						
Mastectomy	1416 ± 855	1393 ± 843	-23 (-226 to 181)	.83	-232 (-612 to 148)	.23
Lumpectomy	1562 ± 1074	1536 ± 936	-26 (-451 to 399)	.90		
Relative lower body endurance (kg/kg)						
Mastectomy	18.60 ± 9.27	19.15 ± 11.81	0.55 (-2.28 to 3.38)	.70	-1.72 (-6.81 to 3.37)	.50
Lumpectomy	21.82 ± 17.23	20.24 ± 11.58	-1.58 (-7.16 to 4.01)	.57		

[a]Adjusted for age, comorbidity, family history of breast cancer, cancer stage, menopausal status, kilocalorie intake, study location, smoking, baseline value of the outcome, treatment status at baseline, other treatment modalities, and reconstruction surgery. Sample sizes: Mastectomy n=73, lumpectomy n=31

Table S15. Association of breast cancer surgery type with change in body composition at 1 year in patients in the AMBER cohort study who received neoadjuvant therapy.

Variable	Baseline	One-year	Baseline to one-year			
			Within-group change		Between-group difference[a]	
	Mean ± SD	Mean ± SD	Mean change (95%CI)	P	Mean difference (95%CI)	P
Body weight (kg)						
Mastectomy	74.4 ± 13.9	73.7 ± 13.9	-0.7 (-2.0 to 0.5)	.23	-1.1 (-3.4 to 1.2)	.36
Lumpectomy	76.7 ± 20.4	77.2 ± 22.3	0.6 (-1.5 to 2.6)	.58		
Body mass index (kg/m^2)						
Mastectomy	27.8 ± 5.4	27.5 ± 5.3	-0.3 (-0.7 to 0.1)	.19	-0.2 (-1.1 to 0.6)	.57
Lumpectomy	27.7 ± 6.4	27.7 ± 6.9	0.0 (-0.8 to 0.8)	.99		
Total lean mass (kg)						
Mastectomy	37.8 ± 4.5	37.0 ± 4.2	-0.7 (-1.3 to -0.2)	.005	-1.1 (-2.1 to -0.1)	.032
Lumpectomy	39.7 ± 6.6	39.7 ± 6.6	0.0 (-0.6 to 0.5)	.98		
Total fat mass (kg)						
Mastectomy	32.4 ± 10.9	32.2 ± 10.7	-0.1 (-1.2 to 1.0)	.83	-0.8 (-2.8 to 1.2)	.43
Lumpectomy	32.4 ± 14.6	33.4 ± 16.1	1.0 (-0.8 to 2.7)	.28		
Lean mass percentage (%)						
Mastectomy	53.0 ± 6.3	52.7 ± 6.3	-0.4 (-1.2 to 0.4)	.35	-0.3 (-1.8 to 1.3)	.75
Lumpectomy	54.5 ± 6.4	54.1 ± 7.1	-0.4 (-1.9 to 1.1)	.60		
Body fat percentage (%)						
Mastectomy	43.7 ± 6.9	44.1 ± 6.7	0.4 (-0.5 to 1.3)	.35	0.2 (-1.4 to 1.9)	.80
Lumpectomy	42.1 ± 6.9	42.6 ± 7.5	0.5 (-1.1 to 2.1)	.53		
Lean-to-fat ratio						
Mastectomy	1.27 ± 0.39	1.25 ± 0.38	-0.02 (-0.08 to 0.03)	.41	-0.03 (-0.14 to 0.09)	.64
Lumpectomy	1.36 ± 0.43	1.35 ± 0.46	-0.01 (-0.12 to 0.10)	.82		
Bone mineral density (g/cm^2)						
Mastectomy	1.16 ± 0.12	1.15 ± 0.12	-0.01 (-0.02 to 0.00)	.011	0.01 (-0.01 to 0.02)	.43
Lumpectomy	1.18 ± 0.10	1.16 ± 0.10	-0.02 (-0.03 to -0.01)	<.001		
Bone mineral content (kg)						
Mastectomy	2.33 ± 0.33	2.28 ± 0.34	-0.05 (-0.07 to -0.03)	<.001	0.00 (-0.05 to 0.04)	.85
Lumpectomy	2.43 ± 0.31	2.38 ± 0.32	-0.06 (-0.08 to -0.03)	<.001		

[a]Adjusted for age, comorbidity, family history of breast cancer, cancer stage, menopausal status, kilocalorie intake, study location, smoking, baseline value of the outcome, treatment status at baseline, other treatment modalities, and reconstruction surgery. Sample sizes: Mastectomy n=73, lumpectomy n=31

Table S16. Association of common treatment combinations with change in health-related fitness at 1 year in the AMBER cohort study, (N=1350)

Variable	Mean change from baseline to one-year (95%CI)			P value
	SCRH (n=392)	SRH (n=313)	SH (n=148)	
Aerobic fitness				
Relative VO_{2peak} (mL/kg/min)	-0.6 (-1.0 to -0.1)	0.0 (-0.5 to 0.6)	0.5 (-0.4 to 1.4)	.14
Absolute VO_{2peak} (L/min)	-0.04 (-0.08 to -0.01)	0.00 (-0.04 to 0.04)	0.07 (0.00 to 0.13)	.022
Muscular strength				
Upper body strength (kg)	0.6 (-0.4 to 1.5)	1.5 (0.4 to 2.6)	2.4 (0.7 to 4.2)	.21
Relative upper body strength (kg/kg)	0.01 (0.00 to 0.03)	0.02 (0.01 to 0.04)	0.02 (0.00 to 0.04)	.75
Lower body strength (kg)	2.6 (-0.4 to 5.5)	2.3 (-1.0 to 5.6)	7.8 (2.4 to 13.2)	.24
Relative lower body strength (kg/kg)	0.05 (0.01 to 0.09)	0.03 (-0.02 to 0.08)	0.08 (0.01 to 0.16)	.53
Muscular endurance				
Upper body endurance (kg)	-8 (-29 to 14)	0 (-24 to 24)	40 (1 to 79)	.15
Relative upper body endurance (kg/kg)	-0.07 (-0.37 to 0.24)	0.03 (-0.32 to 0.37)	0.34 (-0.22 to 0.90)	.51
Lower body endurance (kg)	-118 (-191 to -44)	-3 (-85 to 79)	22 (-112 to 155)	.11
Relative lower body endurance (kg/kg)	-1.48 (-2.48 to -0.47)	-0.20 (-1.32 to 0.92)	0.01 (-1.82 to 1.84)	.24
Body Composition				
Body weight (kg)	-0.4 (-1.2 to 0.3)	-0.1 (-0.9 to 0.7)	1.5 (0.2 to 2.8)	.050
Body mass index (kg/m^2)	-0.1 (-0.4 to 0.2)	-0.1 (-0.4 to 0.2)	0.4 (-0.1 to 1.0)	.21
Total lean mass (kg)	-0.4 (-0.7 to -0.1)	0.0 (-0.4 to 0.3)	0.9 (0.4 to 1.5)	.001
Total fat mass (kg)	0.2 (-0.4 to 0.9)	0.4 (-0.3 to 1.1)	0.7 (-0.4 to 1.8)	.80
Lean mass percentage (%)	-0.4 (-0.8 to 0.1)	-0.4 (-0.9 to 0.1)	0.4 (-0.4 to 1.2)	.29
Body fat percentage (%)	0.5 (0.0 to 1.0)	0.4 (-0.2 to 0.9)	-0.2 (-1.1 to 0.6)	.41
Lean-to-fat ratio	-0.03 (-0.06 to 0.00)	-0.02 (-0.06 to 0.01)	0.01 (-0.05 to 0.07)	.52
Bone mineral density (g/cm^2)	-0.01 (-0.02 to -0.01)	0.00 (-0.01 to 0.01)	0.00 (-0.01 to 0.01)	.052
Bone mineral content (kg)	-0.04 (-0.06 to -0.03)	-0.01 (-0.03 to 0.00)	0.01 (-0.02 to 0.04)	.010

Adjusted for age, comorbidity, family history of breast cancer, cancer stage, menopausal status, kilocalorie intake, study location, smoking, baseline value of the outcome, treatment status at baseline, surgery type, and reconstruction surgery. p-value: tests whether there are significant differences across the various treatment combinations for each HRF component

SCRH: surgery, chemotherapy, radiotherapy, and hormone therapy

SRH: surgery, radiotherapy, and hormone therapy

SH: surgery and hormone therapy

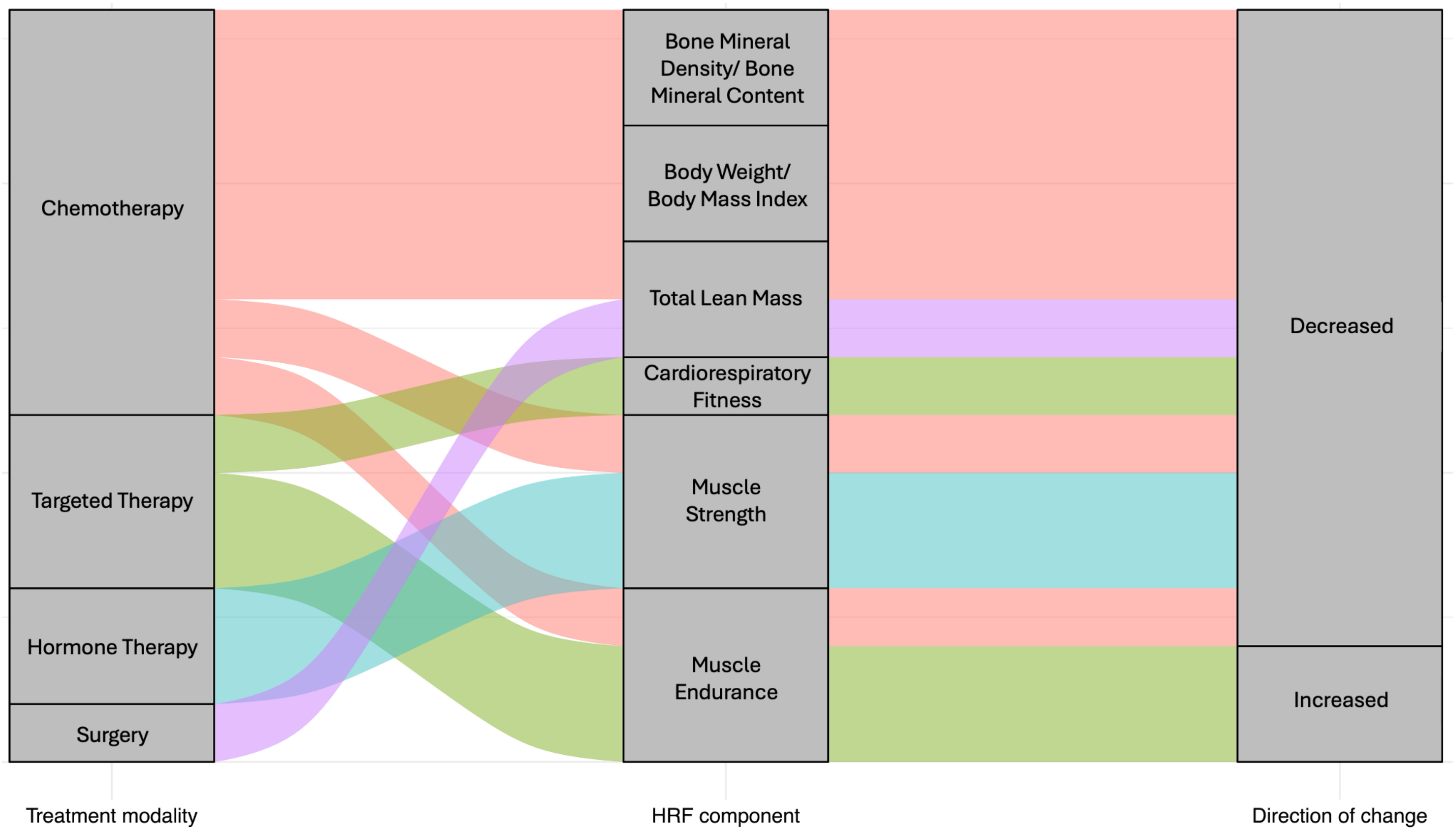

Figure S1. Associations between the individual breast cancer treatment modalities and health-related fitness changes from baseline to 1-year in newly diagnosed breast cancer patients.
